# On‐Surface Synthesis of NBN‐Doped Zigzag‐Edged Graphene Nanoribbons

**DOI:** 10.1002/anie.202000488

**Published:** 2020-03-25

**Authors:** Yubin Fu, Huan Yang, Yixuan Gao, Li Huang, Reinhard Berger, Junzhi Liu, Hongliang Lu, Zhihai Cheng, Shixuan Du, Hong‐Jun Gao, Xinliang Feng

**Affiliations:** ^1^ Center for Advancing Electronics Dresden (cfaed) & Faculty of Chemistry and Food Chemistry Technische Universität Dresden 01062 Dresden Germany; ^2^ Institute of Physics and University of Chinese Academy of Sciences Chinese Academy of Sciences Beijing 100190 China; ^3^ Department of Chemistry and State Key Laboratory of Synthetic Chemistry The University of Hong Kong Pokfulam Road Hong Kong China; ^4^ Department of Physics and Beijing Key Laboratory of Optoelectronic Functional Materials & Micro-nano Devices Renmin University of China Beijing 100872 China

**Keywords:** graphene nanoribbons, NBN doping, on-surface synthesis, radical cations, zigzag edges

## Abstract

We report the first bottom‐up synthesis of NBN‐doped zigzag‐edged GNRs (NBN‐ZGNR1 and NBN‐ZGNR2) through surface‐assisted polymerization and cyclodehydrogenation based on two U‐shaped molecular precursors with an NBN unit preinstalled at the zigzag edge. The resultant zigzag‐edge topologies of GNRs are elucidated by high‐resolution scanning tunneling microscopy (STM) in combination with noncontact atomic force microscopy (nc‐AFM). Scanning tunneling spectroscopy (STS) measurements and density functional theory (DFT) calculations reveal that the electronic structures of NBN‐ZGNR1 and NBN‐ZGNR2 are significantly different from those of their corresponding pristine fully‐carbon‐based ZGNRs. Additionally, DFT calculations predict that the electronic structures of NBN‐ZGNRs can be further tailored to be gapless and metallic through one‐electron oxidation of each NBN unit into the corresponding radical cations. This work reported herein provides a feasible strategy for the synthesis of GNRs with stable zigzag edges yet tunable electronic properties.

## Graphical abstract

**Close to the edge**: Two novel NBN‐doped zigzag‐edged graphene nanoribbons (ZGNRs) are derived through surface‐assisted synthesis. Experiments and calculations reveal the importance of the NBN units in modulating the electronic structures of the ZGNRs. Moreover, theoretical calculations predict that the electronic structures of NBN‐ZGNRs can be further tailored through one‐electron oxidation of each NBN unit into the corresponding radical cations.

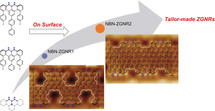

Atomically precise graphene nanoribbons (GNRs) have attracted intense interest due to their fascinating electronic and magnetic properties, which enable next‐generation materials for carbon‐based nanoelectronics and spintronics.^1^ Bottom‐up synthetic strategies, including solution synthesis, chemical vapor deposition (CVD), and surface‐assisted synthesis, represent the most powerful approaches for fabricating GNRs with uniform widths and defined edge structures.^2^ Compared with the solution‐synthesis and CVD‐growth methods, the on‐surface‐synthesis approach takes advantage of in situ characterization of the prepared GNRs and produces high‐quality GNRs that can serve as ideal objects for fundamental studies of graphene‐based electronic devices.^3^ In recent years, a series of GNRs with different edge topologies and widths have been achieved through surface‐assisted synthesis. These edge topologies include armchair, chevron, cove, and zigzag.1a, 4 Compared to armchair‐edged or cove‐edged GNRs (AGNRs or CGNRs), zigzag‐edged GNRs (ZGNRs) are predicted to preserve gapless (zero band gap) or metallic band structures^5^ as well as host spin‐polarized electronic edge states, which render them promising materials in spintronics.4b, 6 Nevertheless, these fascinating properties have barely been observed experimentally due to the lack of a facile synthetic approach and the poor stability of the ZGNRs.^7^ In 2016, the first atomically precise 6‐ZGNR (Figure 1 a) was synthesized on an Au(111) substrate under ultrahigh vacuum (UHV) conditions based on a U‐shaped dibromo dibenzo[*a*,*j*]anthracene monomer (DBBT in Figure 1 a) with a preinstalled zigzag edge and two additional methyl groups at the periphery, which led to the formation of fully zigzag‐edged GNRs by oxidative ring closure of the methyl groups. Scanning tunneling spectroscopy (STS) measurements demonstrated the existence of localized edge states with a large energy splitting at the zigzag edges.4b On the other hand, partial ZGNRs have also raised substantial interest since they can not only enhance the stability but also tune their electronic and magnetic properties, and among them, the most prominent examples exhibit quasi‐one‐dimensional trivial and nontrivial electronic quantum phases.3c, 8 Moreover, heteroatom doping, such as the incorporation of boron and oxygen/nitrogen atoms, at the edges or the central sp^2^‐carbon frameworks of GNRs has been recently demonstrated to modulate the conductance and valence bands of the ribbons and provide access to stable ZGNRs.^9^

**Figure 1:**
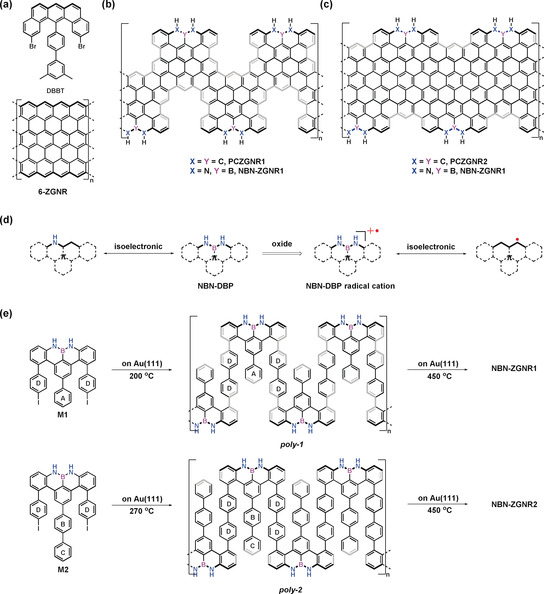
a) Structures of dibromo‐dimethyl‐biphenylbenzo[*m*]tetraphene (DBBT) and 6‐ZGNR.4b b) Structures of pristine fully‐carbon‐based ZGNR1 (PCZGNR1) and NBN‐ZGNR1. c) Structures of PCZGNR2 and NBN‐ZGNR2. d) NBN‐dibenzophenalene (NBN‐DBP) and its radical cation as well as its isoelectronic structures.^10^ e) On‐surface synthetic routes toward NBN‐ZGNR1 and NBN‐ZGNR2.

Among all kinds of heteroatom doping strategies, boron–nitrogen (B–N) doping is particularly interesting due to the isoelectronic and isosteric relationship between C=C and B−N units.^10^ Therefore, substituting a C=C unit in the π‐conjugated system with a B−N subunit can remarkably modulate the electronic structure while maintaining the same conjugated skeleton.^10^, ^11^ In addition to single B−N‐unit doping, substituting a full C_3_ unit on the zigzag edge with a nitrogen–boron–nitrogen (NBN) motif not only provides access to stable zigzag‐edged nanographene (namely, polycyclic aromatic hydrocarbon) but also renders the formation of the radical cation on the NBN edge through selective oxidation, which is the isoelectronic structure of its pristine carbon framework with an open‐shell character (Figure 1 d).^12^ However, the synthesis of NBN‐doped zigzag‐edged GNRs remains challenging due to the lack of a suitable synthetic strategy.

Herein, we report the bottom‐up synthesis of the first NBN‐doped ZGNRs (Figure 1 b,c, NBN‐ZGNR1 and NBN‐ZGNR2) by employing two novel U‐shaped bis(*para*‐iodophenyl)‐substituted NBN‐dibenzophenalene (NBN‐DBP) monomers (M1 and M2, Figure 1 e). The monomers M1 and M2 (with an additional phenyl group at ring A in M1, Figure 1 e) feature the preinstalled zigzag‐edge topologies with two iodo groups at the *para* position of ring D (Figure 1 e), which enables the surface‐assisted polymerization to form swallow‐shaped polymers through thermally induced aryl–aryl coupling (Figure 1 e). Subsequently, intramolecular cyclodehydrogenation of the polymers enables the successful formation of NBN‐doped GNRs with zigzag‐rich edges in which the zigzag‐edge proportions of NBN‐ZGNR1 and NBN‐ZGNR2 are 36 % and 57 %, respectively (for a detailed assignment of the zigzag edges, see Figure S9 in the Supporting Information).3c High‐resolution scanning tunneling microscopy (STM) in combination with noncontact atomic force microscopy (nc‐AFM) clearly reveals the zigzag‐edge topologies of the resultant GNRs. The electronic band gaps of NBN‐ZGNR1 and NBN‐ZGNR2 are determined by scanning tunneling spectroscopy (STS) to be 1.50 eV and 0.90 eV, respectively, which are substantially higher than those of the corresponding pristine carbon‐based ZGNRs (PCZGNR1: 0.52 eV; PCZGNR2: 0.27 eV. Figure 1 b,c). Notably, DFT calculations predict that the electronic structures of the NBN‐doped ZGNRs can be further modulated into gapless or metallic through the one‐electron oxidation of each NBN unit into the radical‐cation form.

The synthetic routes toward the well‐designed U‐shaped monomers M1 and M2 are illustrated in Scheme 1. First, Suzuki coupling was performed between biphenyl diboronic acid pinacol ester **1** and 1‐bromo‐2‐iodo‐3‐nitrobenzene, which provided dibromo‐dinitrophenyl‐biphenyl **4** in 65 % yield. Then, **4** was subjected to Suzuki coupling with (4‐(trimethylsilyl)phenyl)boronic acid to produce dinitrophenyl‐bis(trimethylsilane)‐biphenyl **6** in 71 % yield. Afterwards, the trimethylsilyl (TMS) groups in **6** were converted into iodo groups by treatment with excess iodine monochloride (ICl) to afford diiodophenyl‐dinitrophenyl‐biphenyl **8** in 90 % yield. Subsequently, compound **8** was reduced to diiodophenyl‐diamine‐biphenyl **10** at room temperature under hydrogen gas with Pt/C in 100 % (crude) yield. Finally, heating a solution of **10** in *o*‐dichlorobenzene (*o*‐DCB) at 180 °C in the presence of boron trichloride (BCl_3_) with excess trimethylamine (NEt_3_) gave M1 in 32 % yield. Following a similar synthetic strategy, monomer M2 with an additional phenyl ring at the *para* position of ring A in M1 was successfully synthesized starting from dibromo‐terphenyl **2** over six steps.

**Scheme 1:**
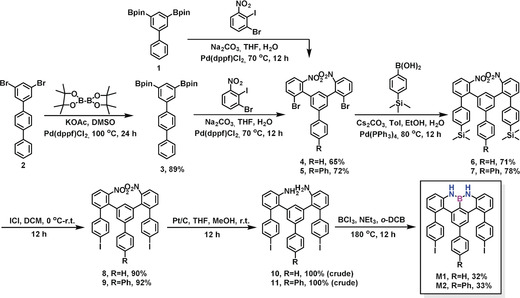
Synthetic routes toward M1 and M2.

The targeted U‐shaped monomers M1 and M2 were purified by silica‐column chromatography and then recrystallized in chloroform/methanol (CHCl_3_/MeOH). Afterwards, M1 and M2 were characterized by high‐resolution matrix‐assisted laser desorption/ionization time‐of‐flight (HR‐MALDI‐TOF) mass spectrometry (Figure 2 a,c). There is only one dominant peak in the respective mass spectrum of M1 and M2, revealing their defined molecular compositions; the isotopic distribution pattern of the mass peak is in good agreement with the calculated pattern (Figure 2 a,c insert). Furthermore, ^1^H NMR spectra of M1 and M2 displayed well‐resolved peaks that could be fully assigned by 2D NMR analysis (Figures 2 b,d and S38–S51). Notably, the singlet peak (orange highlighted peak in Figure 2 b,d) in the ^1^H NMR spectra was assigned to the proton that connects with the nitrogen atom in M1 and M2. Additionally, there is one broad resonance at approximately 27.2 ppm in the ^11^B NMR spectrum of M1 (Figure S41).^13^, ^14^

**Figure 2:**
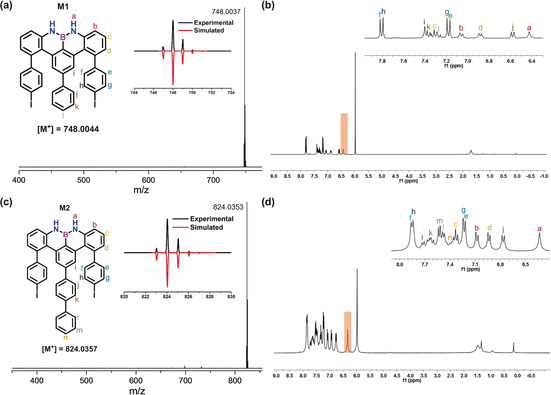
a) HR MALDI‐TOF mass spectrum of M1. b) ^1^H NMR spectrum (300 MHz, 273 K, C_2_D_2_Cl_4_) of M1, insert: assignment for each proton. c) HR MALDI‐TOF mass spectrum of M2. d) ^1^H NMR spectrum (300 MHz, 373 K, C_2_D_2_Cl_4_) of M2, insert: assignment for each proton.

To obtain NBN‐ZGNR1, monomer M1 was sublimed onto an Au(111) substrate held at room temperature under UHV conditions. Constant‐current STM images of the resulting molecular layer revealed that the intact monomers self‐assembled into linear chains (Figure 3 a). Additionally, the assembled structures formed a tail‐to‐tail pattern caused by I⋅⋅⋅H interactions between two monomers (Figure S1). Annealing the substrate at 200 °C induced aryl–aryl coupling, which resulted in linear swallow‐shaped polymer *poly‐* **1** through Ullmann‐type coupling of M1 (Figure 1 e). The polymeric chains adsorbed in the fcc region of the Au(111) reconstruction (Figures 3 b and S2). A closer view of *poly‐* **1** indicated bright protrusions along the chain, which resulted from the rotation of the benzene rings (benzene rings D and A in Figure 1 e) due to the steric hindrance between the neighboring rings (Figures 3 b and S2). Complete cyclodehydrogenation was achieved by further treatment of *poly‐* **1** at 450 °C, which provided fully planarized NBN‐ZGNR1 with an apparent height of 1.75 Å (Figure 3 c). The longest length of the GNRs was up to 30 nm (Figure S6). Additionally, there were some defects caused by the missing benzene ring A in NBN‐ZGNR1 (Figures S3 and S7). These can be attributed to the steric effect between the rotatable middle benzene ring (A) and the side ring (D, Figure 1 e). Therefore, ring A may detach from the polymer at high temperatures before cyclodehydrogenation. Furthermore, the chemical structure of the resultant NBN‐ZGNR1 was unambiguously confirmed by nc‐AFM measurements using a CO‐functionalized tip. Figure 3 d depicts the resulting constant‐height frequency‐shift image where the periodic aromatic carbon atoms together with the nitrogen and boron atoms are clearly unveiled. In the previously reported B‐AGNRs, B,N‐AGNRs, and oxygen–boron–oxygen (OBO) chiral GNRs,9a, 9c, 9f the boron atoms appeared with a darker contrast (more negative frequency shift) due to a stronger interaction with the gold substrate. These contrasting results can be explained by the same interaction of the nitrogen, boron, and carbon atoms with the gold substrate. Notably, NBN‐ZGNR1 was found to laterally align on the Au(111) substrate (Figure 3 c).

**Figure 3:**
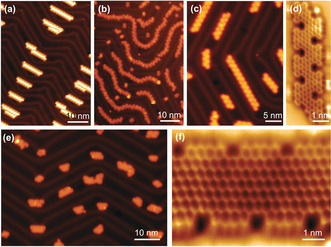
a) STM image of M1 as sublimed on Au(111). b) STM image of M1 after annealing at 200 °C on Au(111), inducing deiodination and polymerization. c) STM image of M1 after annealing at 450 °C on Au(111). d) nc‐AFM image of NBN‐ZGNR1. e) STM image of M2 after annealing at 450 °C on Au(111), showing the formation of NBN‐ZGNR2. f) nc‐AFM image of NBN‐ZGNR2. Scanning parameters: (a)–(c) and (e) *V*=−500 mV and I=30 pA. (d,f) amplitude=100 pm.

Following a similar synthetic procedure, NBN‐ZGNR2 was successfully synthesized from monomer M2 on the surface. M2 was sublimed onto a Au(111) substrate and held at room temperature under UHV conditions, where it formed self‐assembled linear chains (Figure S4). Then, linear swallow‐shaped polymer *poly‐* **2** was formed after annealing the substrate at 270 °C through Ullmann‐type coupling (see Figure S4). Finally, NBN‐ZGNR2 was achieved by annealing *poly‐* **2** at 450 °C, which exhibited an apparent height of 1.75 Å (Figure 3 e), and the longest length was up to 12.3 nm (Figure S5). Due to the increased steric hindrance between the central benzene rings (B and C) and the side rings (D, Figure 1 e) in the precursor M2 and *poly‐* **2**, the resultant length of NBN‐ZGNR2 is shorter than that of NBN‐ZGNR1. From a statistical analysis (Figure S6), the length of most NBN‐ZGNR2 (or NBN‐ZGNR2 segments) is less than 7 nm (nine repeating units). Similar to NBN‐ZGNR1, the nitrogen and boron atoms were clearly unveiled in NBN‐ZGNR2 by nc‐AFM measurements (Figure 3 f). Due to the NBN edge doping, these two GNRs featured stable zigzag edges in which no further reaction such as edge fusing was observed after thermal annealing, in contrast to the pristine carbon‐based ZGNRs.3c

To gain insight into the electronic properties of NBN‐ZGNRs, differential conductance (d*I*/d*V*) spectra (Figure 4) based on these two GNRs were probed on Au(111). The conduction band (CB) and valence band (VB) could be affected by the electronic states of the substrate and only appear like onsets in the STS measurement.^15^ The CB and VB of NBN‐ZGNR1 were identified as the onset of the electronic bands, which are located at 0.50 eV and −1.00 eV (Figure 4 a), respectively. Its corresponding electronic band gap is derived to be 1.50 eV. From the DFT calculations (Figure 4 b), the band gap of NBN‐ZGNR1 is estimated to be 1.53 eV, which is in line with the experimental STS result. Additionally, the d*I*/d*V* spectra in Figure 4 c reveal the CB and VB of the NBN‐ZGNR2 segment (six repeating units) to be located at 0.20 eV and −0.70 eV, respectively. Accordingly, the corresponding electronic band gap is 0.90 eV, which is further supported by DFT calculations (Figure 4 d, 0.84 eV). The band gap of NBN‐ZGNR2 (0.90 eV) is clearly smaller than that of NBN‐ZGNR1 (1.50 eV), which can be attributed to the laterally expanded width of the GNR with a large π‐conjugated system. In contrast to the pure‐carbon‐based full ZGNRs, which have a zero band gap,^5^ the resultant NBN‐doped ZGNRs possess a defined band gap, which is comparable to those of 13‐AGNR (1.40 eV)3b and 15‐AGNR (0.86 eV).^16^, ^17^ On the other hand, DFT calculations predict that NBN‐ZGNR1 (1.53 eV) and NBN‐ZGNR2 (0.84 eV) exhibit larger band gaps than their corresponding pristine‐carbon‐based ZGNRs (PCZGNR1: 0.52 eV, Figure 5 a; PCZGNR2: 0.27 eV, Figure 5 b; see the structures in Figure 1 b,c).

**Figure 4:**
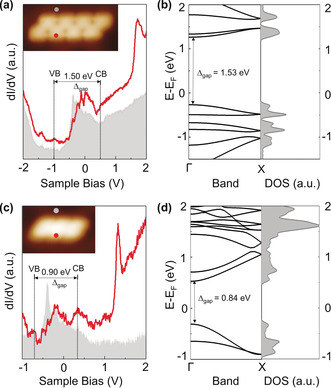
STS and calculated band structures of NBN‐ZGNR1 and NBN‐ZGNR2. a) Differential conductance (d*I*/d*V*) spectra taken on NBN‐ZGNR1, CB: conduction band, VB: valence band. b) DFT‐calculated band structure and density of states (DOS) results of NBN‐ZGNR1. c) Differential conductance (d*I*/d*V*) spectra taken on NBN‐ZGNR2. d) DFT‐calculated band structure and DOS results of NBN‐ZGNR2.

**Figure 5:**
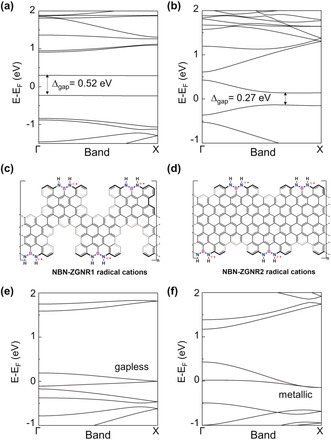
a), b) DFT‐calculated band structures of pristine PCZGNR1 and PCZGNR2. The bands of different spins are degenerated. c), d) Chemical structures of NBN‐ZGNR1 and NBN‐ZGNR2 radical cations. e), f) DFT‐calculated band structures of NBN‐ZGNR1 radical cations and NBN‐ZGNR2 radical cations in which each NBN unit loses one electron. The bands of different spins are degenerated.

As the NBN motif on the zigzag edge can be further oxidized into the corresponding radical cation, which is the isoelectronic structure to its pristine carbon framework (Figure 1 d), this enables a potential chemical tunability of NBN‐ZGNRs. To this end, DFT calculations were carried out to study the electronic structures of NBN‐ZGNRs after one‐electron oxidation for each NBN unit (Figure 5). Interestingly, when NBN‐ZGNR1 and NBN‐ZGNR2 are oxidized into radical cations (Figure 5 c,d, each NBN unit loses one electron), their band structures become gapless (0 eV) and metallic, respectively (Figure 5 e,f). Compared to the pristine carbon‐based PCZGNR1 (0.52 eV) and PCZGNR2 (0.27 eV) (Figure 5 a,b), which are low‐band‐gap semiconductors, NBN‐ZGNRs with radical cations clearly show different electronic structures.

In summary, we demonstrated the bottom‐up on‐surface synthesis of the first NBN‐doped ZGNRs, which are derived from two novel U‐shaped molecular precursors with preinstalled NBN zigzag edges. The geometric structures of the zigzag topologies of NBN‐doped ZGNRs have been unambiguously characterized by STM and nc‐AFM. STS analysis together with DFT calculations revealed that the NBN units play a significant role in modulating the electronic structures of ZGNRs (NBN‐ZGNR1: 1.50 eV; NBN‐ZGNR2: 0.90 eV) compared with those of their corresponding pristine carbon‐based ZGNRs (PCZGNR1: 0.52 eV; PCZGNR2: 0.27 eV). Moreover, theoretical calculations predicted that the band structures of NBN‐ZGNRs can be further tailored to be gapless or metallic through the selective oxidation of the NBN units into the formation of radical cations. The synthetic strategy established in this work provides an opportunity to fabricate stable GNRs containing zigzag edges and lends credence to their possible applications in graphene‐based nanoelectronic devices. Moreover, the chemical tunability of NBN‐ZGNRs paves the way for investigating the isoelectronic structures of pure‐carbon‐based ZGNRs.

## Conflict of interest

The authors declare no conflict of interest.

## Supporting information

As a service to our authors and readers, this journal provides supporting information supplied by the authors. Such materials are peer reviewed and may be re‐organized for online delivery, but are not copy‐edited or typeset. Technical support issues arising from supporting information (other than missing files) should be addressed to the authors.

Supplementary
