# Supplementary material for: On‐Surface Synthesis of NBN‐Doped Zigzag‐Edged Graphene Nanoribbons
Source: Angew Chem Int Ed Engl. 2020 Mar 25;59(23):8873–9. doi: 10.1002/anie.202000488 (PMC7318338; doi:10.1002/anie.202000488)
Supplement: Supplementary file 1 — Supplementary [file ANIE-59-8873-s001.pdf]

Angewandte Chemie
Eine Zeitschrift der Gesellschaft Deutscher Chemiker

Supporting Information

# On-Surface Synthesis of NBN-Doped Zigzag-Edged Graphene Nanoribbons

*Yubin Fu[+], Huan Yang[+], Yixuan Gao[+], Li Huang,* Reinhard Berger, Junzhi Liu,* Hongliang Lu, Zhihai Cheng, Shixuan Du, Hong-Jun Gao, and Xinliang Feng**

anie_202000488_sm_miscellaneous_information.pdf

## *Contents*

1. Experimental details 1

2. Synthetic procedures 3

3. STM results 15

4. Assignment of zigzag edge proportion 19

5. Details of the theoretical calculations 20

6. NMR spectra 21

7. High-Resolution Mass Spectroscopy (HRMS) 42

8. References 43

## 1. Experimental details

General methods and materials: 3,5-bis(4,4,5,5-tetramethyl-1,3,2-dioxaborolan-2-yl)-1,1'-biphenyl (**1**),[1] 3,5-dibromo-1,1':4',1"-terphenyl (**2**),[2] and 1-bromo-2-iodo-3-nitrobenzene[3] were prepared as the reported methods. All the other reagents were obtained from Sigma Aldrich, TCI, abcr, Alfa Aesar and Strem. All these chemicals were used as received without further purification. Anhydrous toluene, tetrahydrofuran (THF) and dichloromethane (DCM) were obtained from MBRAUN MB-SPS-5 solvent purification system and anhydrous dimethyl sulfoxide (DMSO) *o*-dichlorobenzene (*o*-DCB) was obtained from Acros organics. All the sensitive reactions were performed using standard vacuum-line and Schlenk techniques. Thin layer chromatography (TLC) was performed on silica-coated aluminum sheets with a fluorescence indicator (TLC silica gel 60 $F_{254}$, purchased from Merck KGaA). Column chromatography was performed on silica ($SiO_2$, particle size 0.063–0.200 mm, purchased from VWR). NMR spectra were recorded on a Bruker AV-II 300 spectrometer operating at 300 MHz for $^1$H, 76 MHz for $^{13}$C and 96 MHz for $^{11}$B with standard Bruker pulse programs at room temperature (296 K). $^{11}$B NMR chemical shifts were referenced to the external standard boron signal of boron trifluoride etherate ($BF_3 \cdot Et_2O$) ($\delta$ = 0 ppm). Dichloromethane-d$_2$ ($CD_2Cl_2$) ($^1$H, $\delta$ = 5.32 ppm, $^{13}$C, $\delta$ = 53.8 ppm) or 1,1,2,2-tetrachloroethane-d$_2$ ($C_2D_2Cl_4$) ($^1$H, $\delta$ = 5.98 ppm; $^{13}$C, $\delta$ = 74.4 ppm) was used as solvent and tetramethylsilane (TMS) ($\delta_{TMS}$ = 0.00) was used as internal standard. The following abbreviations are used to describe peak patterns as appropriate: s = singlet, d = doublet, t = triplet, q = quartet, and m = multiplet. The mass spectrometry analysis was performed on a Bruker Autoflex Speed MALDI TOF MS (Bruker Daltonics, Bremen, Germany) using DCTB (trans-2-[3-(4-tert-Butylphenyl)-2-methyl-2-propenylidene]malononitrile) as matrix or Agilent Q-TOF (APCI mode using acetonitrile as solvent) instruments.

On-surface experiments were performed on a CreaTec ultra-high vacuum STM/nc-AFM system (STM: scanning tunneling microscopy; nc-AFM: non-contact atomic force microscopy) with a base pressure of $2 \times 10^{-10}$ mbar. The Au (111) substrate was prepared by repeated cycles of Ar+ ion sputtering and annealing at 700 K for 20 min. The monomers M1 and M2 were sublimed onto clean Au(111) surface at 520 K and 540 K, respectively, while keeping the substrate at room temperature. All STM and nc-AFM measurements were performed with a Pt/Ir tip at LHe temperature. The STM images were taken in the constant current mode. The nc-AFM measurements were performed using a commercial qPlus tuning fork sensor[4] in

frequency modulation mode with a CO-terminated Pt/Ir tip.[5] The sensor was driven at its resonance frequency of 27.9 kHz with a constant amplitude of 100 pm. The shift in the resonance frequency of the tuning fork was recorded in the constant-height mode. The STM and nc-AFM images were analyzed using WSxM. d$I$/d$V$ spectra were measured using a lock-in technique with a sinusoidal modulation of 10 mV rms at a frequency of 973 Hz. The tips were calibrated on clean Au(111) surface before spectroscopic measurements to ensure no tip-related features on the recorded d$I$/d$V$ spectra.

## 2. Synthetic procedures

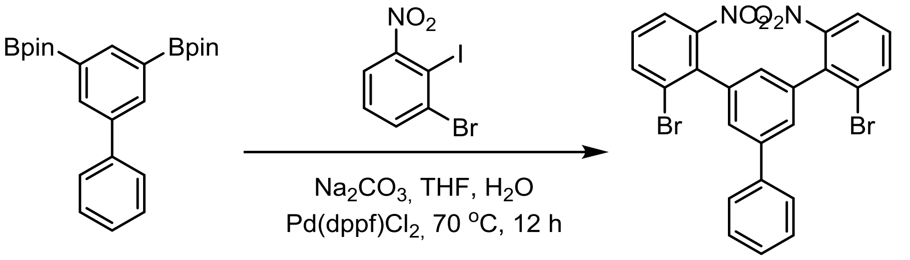


1 4

**2,2''-dibromo-6,6''-dinitro-5'-phenyl-1,1':3',1''-terphenyl (4).** In a 100 mL long-necked Schlenk flask, compound **1** (453.0 mg, 1.11 mmol), 1-bromo-2-iodo-3-nitrobenzene (916.0 mg, 2.79 mmol), $Na_2CO_3$ (1.1924 g, 11.25 mmol) and Pd(dppf)$Cl_2$ (62.5 mg, 0.076 mmol) were charged under the protection of argon. After adding 15 mL degassed THF and $H_2O$ (3:1), the mixture was heated to 70 °C and stirred for 12 hours. After cooling down to room temperature, the reactant was poured into brine and extracted by dichloromethane for three times. The organic phase was dried over magnesium sulfate and the solvent was evaporated in vacuo. The crude product was purified by flash chromatography on silica gel ($CH_2Cl_2$/iso-hexane = 1/2) to give product as light yellow oily liquid, which solidified slowly (399.8 mg, 65 %).

$^1$H NMR (300 MHz, $CD_2Cl_2$) $\delta$ 7.88 (dd, J = 2.4, 1.2 Hz, 1H), 7.85 (dd, J = 2.4, 1.2 Hz, 1H), 7.78 (dd, J = 4.5, 1.2 Hz, 1H), 7.75 (dd, J = 4.5, 1.2 Hz, 1H), 7.58 – 7.54 (m, 1H), 7.53 (dd, J = 1.9, 0.9 Hz, 1H), 7.46 (d, J = 1.6 Hz, 1H), 7.44 (d, J = 1.6 Hz, 1H), 7.40 – 7.24 (m, 5H), 7.05 (dt, J = 8.3, 1.6 Hz, 1H);

$^{13}$C NMR (76 MHz, $CD_2Cl_2$) $\delta$ 150.8, 141.5, 139.5, 137.1, 136.8, 136.7, 135.7, 135.7, 129.8, 129.8, 128.9, 128.5, 128.3, 128.0, 127.4, 127.3, 127.1, 126.0, 125.9, 122.9, 122.8;

Comments: Although compound **4** shows $C_2$ symmetry, its $^{13}$C NMR spectrum exhibits more than 14 peaks (Fig. S15). This is caused by the presence of conformational isomers (the different rotation angle between the middle biphenyl group with the bromo-nitrobenzene group). This result is similar to the previous reported molecules[6-7] and these isomers will not affect the following reactions.

HRMS (MADLI-TOF, m/z): calcd for $C_{24}H_{14}Br_2N_2O_4 + K^+$ $[M+K]^+$ 592.8933, found 592.8931.

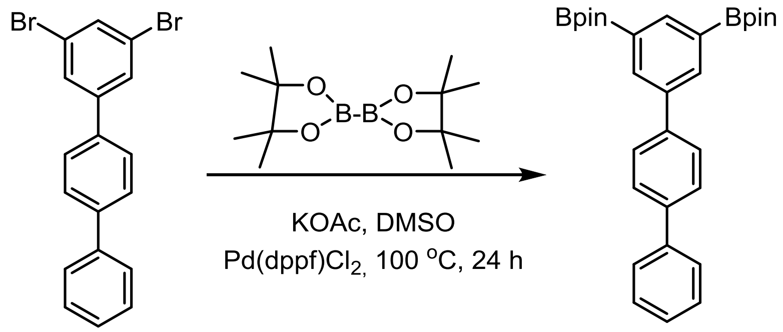


2 3

**3,5-bis(4,4,5,5-tetramethyl-1,3,2-dioxaborolan-2-yl)-1,1':4',1''-terphenyl (3).** In a 200 mL long-necked Schlenk flask, compound **2** (1.9777 g, 5.09 mmol), 4,4,4',4',5,5,5',5'-octamethyl-2,2'-bi(1,3,2-dioxaborolane) (2.8430 g, 11.19 mmol), KOAc (2.6434 g, 26.93 mmol) and Pd(dppf)$Cl_2$ (238.0 mg, 0.29 mmol) were charged under the protection of argon. After adding 35 mL degassed DMSO, the mixture was heated to 100 °C and stirred for 24 hours. After cooling down to room temperature, the reactant was poured into brine and extracted by ethyl acetate for three times. The organic phase was dried over magnesium sulfate and the solvent was evaporated in vacuo. The crude product was purified by flash chromatography on silica gel (ethyl acetate/iso-hexane = 1/2) then recrystallized from n-hexane to give product as white powder (2.1974 g, 89 %).

$^{1}$H NMR (300 MHz, $CD_2Cl_2$) $\delta$ 8.16 (s, 3H), 7.81 – 7.74 (m, 2H), 7.73 – 7.65 (m, 4H), 7.52 – 7.43 (m, 2H), 7.41 – 7.32 (m, 1H), 1.37 (s, 24H);

$^{11}$B NMR (96 MHz, $CD_2Cl_2$) $\delta$ 30.4;

$^{13}$C NMR (76 MHz, $CD_2Cl_2$) $\delta$ 141.0, 140.7, 140.3, 140.2, 139.5, 136.2, 129.2, 127.9, 127.7, 127.7, 127.3, 84.3, 25.1;

Comments: The $^{13}$C NMR signal of the carbon adjacent to boron was not observed (Fig. S11).

HRMS (MADLI-TOF, m/z): calcd for $C_{30}H_{36}B_2O_4^+$ $[M]^+$ 482.2800, found 482.2799.

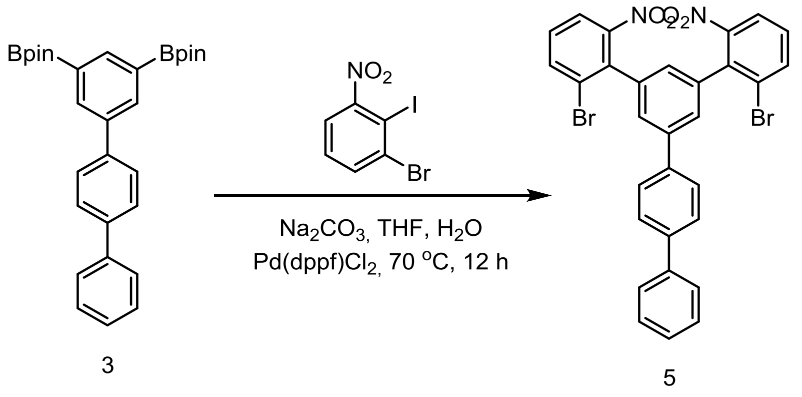


**2-bromo-5'-(2-bromo-6-nitrophenyl)-6-nitro-1,1':3',1'':4'',1'''-quaterphenyl (5).** In a 200 mL long-necked Schlenk flask, compound **3** (490.7 mg, 1.02 mmol), 1-bromo-2-iodo-3-nitrobenzene (1.0293 mg, 3.14 mmol), $Na_2CO_3$ (1.2278 g, 11.58 mmol) and Pd(dppf)$Cl_2$ (82.7 mg, 0.10 mmol) were charged under the protection of argon. After adding 24 mL degassed THF and $H_2O$ (3:1), the mixture was heated to 70 °C and stirred for 12 hours. After cooling down to room temperature, the reactant was poured into brine and extracted by dichloromethane for three times. The organic phase was dried over magnesium sulfate and the solvent was evaporated in vacuo. The crude product was purified by flash chromatography on silica gel ($CH_2Cl_2$/iso-hexane = 1/2) to give product as light yellow oily liquid, which solidified slowly (464.4 mg, 72 %).

$^1$H NMR (300 MHz, $CD_2Cl_2$) $\delta$ 7.99 (dd, J = 2.3, 1.2 Hz, 1H), 7.96 (dd, J = 2.3, 1.2 Hz, 1H), 7.89 (dd, J = 4.4, 1.2 Hz, 1H), 7.86 (dd, J = 4.4, 1.2 Hz, 1H), 7.72 (d, J = 1.5 Hz, 4H), 7.68 (d, J = 1.5 Hz, 1H), 7.65 (s, 1H), 7.61 (d, J = 1.6 Hz, 1H), 7.59 (d, J = 1.6 Hz, 1H), 7.50 – 7.41 (m, 4H), 7.40 – 7.33 (m, 1H), 7.16 (dt, J = 8.3, 1.6 Hz, 1H);

$^{13}$C NMR (75 MHz, $CD_2Cl_2$) $\delta$ 150.8, 141.0, 140.7, 140.3, 138.4, 137.2, 136.8, 136.7, 135.7, 135.6, 129.8, 129.8, 128.8, 128.5, 128.4, 127.5, 127.3, 127.2, 126.9, 126.0, 125.9, 122.9, 122.8;

Comments: Similar to compound **4**, the $^{13}$C NMR spectrum of compound **5** exhibits more than 18 peaks (Fig. S18). This is also caused by the presence of conformational isomers (the different rotation angle between the middle terphenyl group with the bromo-nitrobenzene group). This result is similar to the previous reported molecules[6-7] and these isomers will not affect the following reactions.

HRMS (MADLI-TOF, m/z): calcd for $C_{30}H_{18}Br_2N_2O_4^+$ [M]$^+$ 629.9613, found 629.9607.

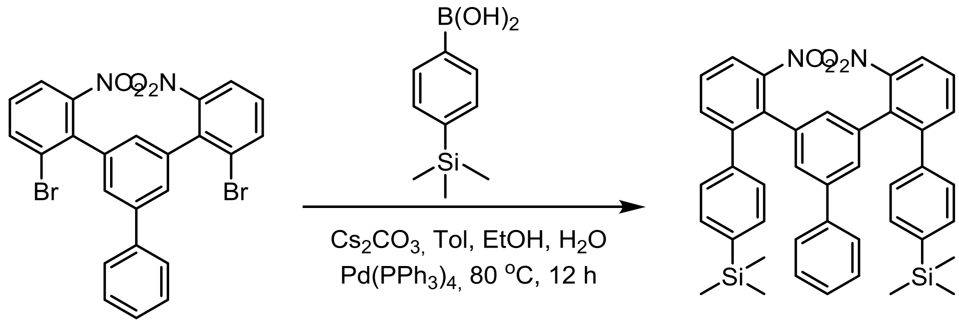

4 6

**(3',6'''-dinitro-5''-phenyl-[1,1':2',1'':3'',1''':2''',1''''-quinquephenyl]-4,4''''-diyl)bis(trimethylsilane) (6).** In a 100 mL long-necked Schlenk flask, compound **4** (227.7 mg, 0.41 mmol), (4-(trimethylsilyl)phenyl)boronic acid (240.4 mg, 1.24 mmol), $Cs_2CO_3$ (1.3745 g, 4.22 mmol) were charged under the protection of argon. After adding 10 mL toluene, ethanol and $H_2O$ (3:1:1), the mixture was degassed by Ar for 30 min. Then $Pd(PPh_3)_4$ (49.0 mg, 0.042 mmol) was charged under the protection of argon and the mixture was heated to 80 °C and stirred for 12 hours. After cooling down to room temperature, the reactant was poured into brine and extracted by dichloromethane for three times. The organic phase was dried over magnesium sulfate and the solvent was evaporated in vacuo. The crude product was purified by flash chromatography on silica gel ($CH_2Cl_2$/iso-hexane = 1/4) to give product as yellow oily liquid, which solidified slowly (185.5 mg, 71 %).

$^1$H NMR (300 MHz, $CD_2Cl_2$) $\delta$ 7.80 (d, $J$ = 8.0 Hz, 2H), 7.69 (d, $J$ = 7.4 Hz, 2H), 7.57 (t, $J$ = 7.7 Hz, 2H), 7.47 (d, $J$ = 7.4 Hz, 4H), 7.22 (d, $J$ = 13.3 Hz, 3H), 7.07 (d, $J$ = 7.6 Hz, 4H), 6.94 (d, $J$ = 5.7 Hz, 2H), 6.86 (s, 2H), 6.61 (d, $J$ = 3.1 Hz, 1H), 0.22 (s, 18H);

$^{13}$C NMR (75 MHz, $CD_2Cl_2$) $\delta$ 151.6, 151.5, 151.1, 151.1, 144.4, 141.7, 140.8, 139.9, 139.8, 136.3, 134.2, 133.6, 133.4, 129.7, 129.0, 128.8, 128.7, 127.8, 127.8, 127.7, 127.6, 127.4, 122.9, -1.1.

Comments: Similar to compound **4**, the $^{13}$C NMR spectrum of compound **6** exhibits more than 20 peaks (Fig. S21). This is also caused by the presence of conformational isomers (the different rotation angle between the middle biphenyl group with the trimethylsilane-nitro-biphenyl group). This result is similar to the previous reported molecules[6-7] and these isomers will not affect the following reactions.

HRMS (ACPI, m/z): calcd for $C_{42}H_{40}N_2O_4Si_2^+$ [M]$^+$ 692.2527, found 692.2524.

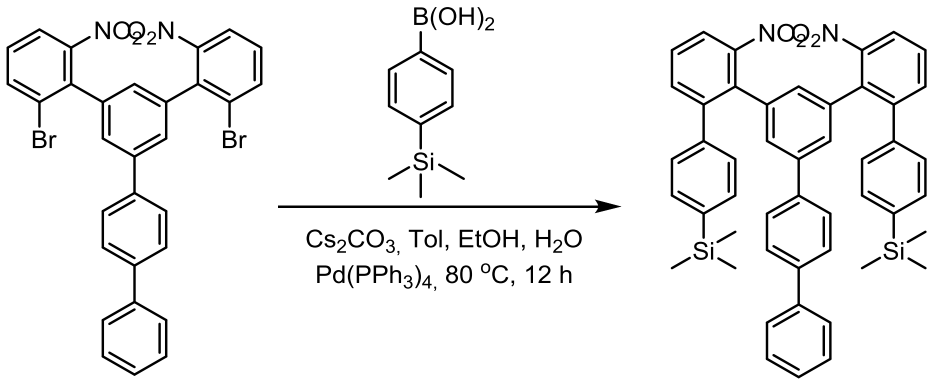

5 7

**(5''-([1,1'-biphenyl]-4-yl)-3',6'''-dinitro-[1,1':2',1'':3'',1''':2''',1''''-quinquephenyl]-4,4''''-diyl)bi-s(trimethylsilane) (7).** In a 200 mL long-necked Schlenk flask, compound **5** (464.4 mg, 0.74 mmol), (4-(trimethylsilyl)phenyl)boronic acid (685.0 mg, 3.53 mmol), $Cs_2CO_3$ (2.4217 g, 7.43 mmol) were charged under the protection of argon. After adding 20 mL toluene, ethanol and $H_2O$ (3:1:1), the mixture was degassed by Ar for 30 min. Then $Pd(PPh_3)_4$ (118.5 mg, 0.10 mmol) was charged under the protection of argon and the mixture was heated to 80 °C and stirred for 12 hours. After cooling down to room temperature, the reactant was poured into brine and extracted by dichloromethane for three times. The organic phase was dried over magnesium sulfate and the solvent was evaporated in vacuo. The crude product was purified by flash chromatography on silica gel ($CH_2Cl_2$/iso-hexane = 1/4) to give product as yellow oily liquid, which solidified slowly (443.2 mg, 78 %).

$^1$H NMR (300 MHz, $CD_2Cl_2$) $\delta$ 7.82 (d, $J$ = 7.8 Hz, 2H), 7.71 (d, $J$ = 7.4 Hz, 1H), 7.64 – 7.53 (m, 5H), 7.53 – 7.45 (m, 6H), 7.43 (d, $J$ = 7.7 Hz, 2H), 7.38 – 7.29 (m, 2H), 7.11 (d, $J$ = 7.7 Hz, 4H), 6.93 (t, $J$ = 13.0 Hz, 4H), 6.74 (d, $J$ = 7.7 Hz, 1H), 0.24 (s, 18H);

$^{13}$C NMR (76 MHz, $CD_2Cl_2$) $\delta$ 151.6, 151.1, 144.4, 143.9, 141.1, 140.8, 140.4, 140.1, 139.9, 139.8, 136.4, 136.1, 134.2, 133.6, 133.4, 133.1, 133.1, 129.7, 129.4, 129.2, 128.8, 128.6, 127.8, 127.8, 127.4, 127.2, 122.9, -1.1.

Comments: Similar to compound **4**, the $^{13}$C NMR spectrum of compound **7** exhibits more than 24 peaks (Fig. S24). This is also caused by the presence of conformational isomers (the different rotation angle between the middle terphenyl group with the trimethylsilane-nitro-biphenyl group). This result is similar to the previous reported molecules[6-7] and these isomers will not affect the following reactions.

HRMS (ACPI, m/z): calcd. for $C_{48}H_{44}N_2O_4Si_2^+$ [M+H]$^+$ 768.2840, found 768.2830.

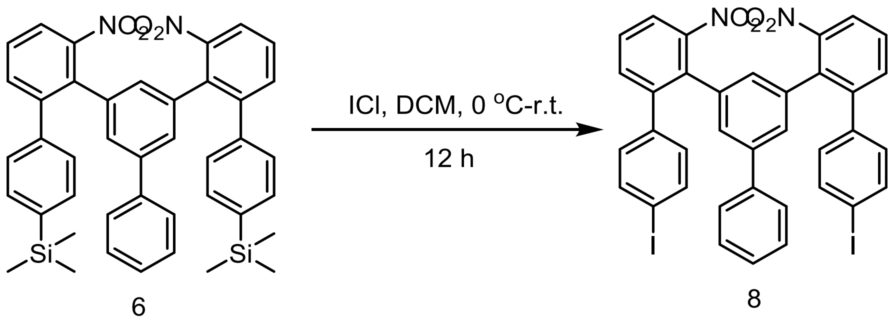

**4,4''''-diiodo-3',6'''-dinitro-5''-phenyl-1,1':2',1'':3'',1''':2''',1''''-quinquephenyl (8).** In a 50 mL long-necked Schlenk flask, compound **6** (185.5 mg, 0.30 mmol) was charged under the protection of argon. After adding 9.5 mL anhydrous DCM, the mixture was cooled to 0 °C. ICl (281.0 mg, 1.73 mmol, dissolved in 1.5 mL anhydrous DCM) was charged under the protection of argon and the mixture was stirred for 10 minutes. Then the ice bath was removed and the resulting mixture was stirred at room temperature for 12 hours. Afterwards the reaction was quenched with 20 mL saturated sodium sulfite solution, and extracted by dichloromethane for three times. The organic phase was dried over magnesium sulfate and the solvent was evaporated in vacuo. The crude product was purified by flash chromatography on silica gel ($CH_2Cl_2$/iso-hexane = 1/2) to give product as light yellow solid (213.3 mg, 90 %).

$^1$H NMR (300 MHz, $CD_2Cl_2$) $\delta$ 7.82 (d, $J$ = 7.1 Hz, 2H), 7.69 – 7.62 (m, 5H), 7.59 (t, $J$ = 7.8 Hz, 3H), 7.30 (d, $J$ = 6.2 Hz, 3H), 7.10 (d, $J$ = 12.8 Hz, 1H), 7.00 (d, $J$ = 13.4 Hz, 2H), 6.94 – 6.87 (m, 2H), 6.83 (t, $J$ = 10.3 Hz, 3H), 6.70 (d, $J$ = 6.0 Hz, 1H);

$^{13}$C NMR (76 MHz, $CD_2Cl_2$) $\delta$ 151.1, 143.2, 141.9, 140.2, 138.9, 137.7, 137.6, 136.3, 134.0, 134.0, 133.4, 132.3, 132.1, 130.9, 129.3, 129.1, 129.0, 128.6, 128.5, 128.3, 128.0, 127.3, 123.1, 93.8.

Comments: Similar to compound **4**, the $^{13}$C NMR spectrum of compound **8** exhibits more than 20 peaks (Fig. S27). This is also caused by the presence of conformational isomers (the different rotation angle between the middle biphenyl group with the iodo-nitro-biphenyl group). This result is similar to the previous reported molecules[6-7] and these isomers will not affect the following reactions.

HRMS (ACPI, m/z): calcd for $C_{36}H_{22}I_2N_2O_4^+$ $[M+H]^+$ 799.9669, found 799.9656.

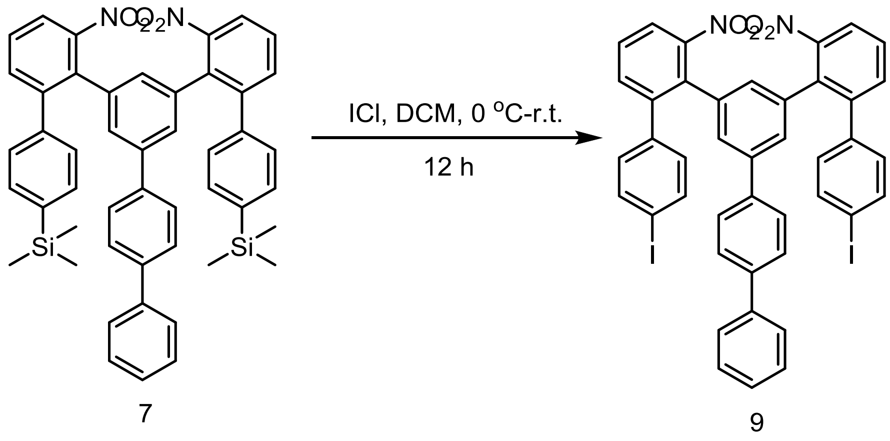

**5''-([1,1'-biphenyl]-4-yl)-4,4''''-diiodo-3',6'''-dinitro-1,1':2',1'':3'',1''':2''',1''''-quinquephenyl (9).** In a 100 mL long-necked Schlenk flask, compound **7** (443.2 mg, 0.58 mmol) was charged under the protection of argon. After adding 20 mL anhydrous DCM the mixture was cooled to 0 $^{o}$C. ICl (555.6 mg, 3.42 mmol, dissolved in 3 mL anhydrous DCM) was charged under the protection of argon and the mixture was stirred for 10 minutes. Then the ice bath was removed and the resulting mixture was stirred at room temperature for 12 hours. Afterwards the reaction was quenched with 50 mL saturated sodium sulfite solution, and extracted by dichloromethane for three times. The organic phase was dried over magnesium sulfate and the solvent was evaporated in vacuo. The crude product was purified by flash chromatography on silica gel ($CH_2Cl_2$/iso-hexane = 1/2) to give product as light yellow solid (428.3 mg, 92 %).

$^{1}$H NMR (300 MHz, $CD_2Cl_2$) $\delta$ 7.87 – 7.80 (m, 2H), 7.77 (d, $J$ = 8.4 Hz, 1H), 7.66 (d, $J$ = 7.5 Hz, 5H), 7.57 (dd, $J$ = 18.9, 10.8 Hz, 6H), 7.49 – 7.39 (m, 2H), 7.33 (dd, $J$ = 13.0, 7.3 Hz, 1H), 7.04 (t, $J$ = 19.6 Hz, 5H), 6.86 (d, $J$ = 7.7 Hz, 3H), 6.71 (d, $J$ = 5.9 Hz, 1H);

$^{13}$C NMR (76 MHz, $CD_2Cl_2$) $\delta$ 151.1, 143.2, 140.7, 140.3, 139.4, 138.9, 138.2, 137.7, 136.3, 134.0, 133.4, 132.1, 131.7, 130.3, 130.2, 129.2, 129.2, 129.1, 129.1, 128.6, 128.5, 127.8, 127.7, 127.5, 127.4, 127.3, 123.1, 93.8, 93.4.

Comments: Similar to compound **4**, the $^{13}$C NMR spectrum of compound **9** exhibits more than 24 peaks (Fig. S30). This is also caused by the presence of conformational isomers (the different rotation angle between the middle terphenyl group with the iodo-nitro-biphenyl group). This result is similar to the previous reported molecules[6-7] and these isomers will not affect the following reactions.

HRMS (ACPI, m/z): calcd for $C_{42}H_{26}I_2N_2O_4^+$ [M]$^+$ 875.9982, found 875.9970.

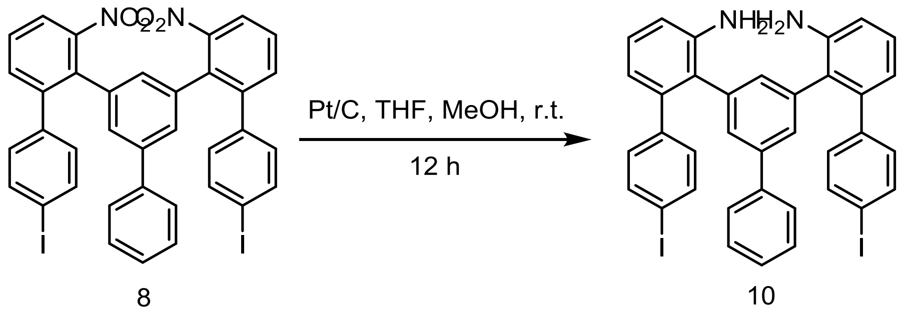


**4,4''''-diiodo-5''-phenyl-[1,1':2',1'':3'',1''':2''',1''''-quinquephenyl]-3',6'''-diamine (10).** In a 50 mL one-necked flask, compound **8** (213.3 mg, 0.27 mmol) was charged under the protection of argon. Then 12 mL THF and methanol (1:1) was added into the flask. After Pt/C (42.0 mg, 0.21 mmol) was charged under the protection of argon, the mixture was degassed by $H_2$ for 5 minutes. And the resulting mixture was stirred at room temperature for 12 hours with a $H_2$ balloon. Afterwards the mixture was filtered by celite and the solvent was evaporated in vacuo. The crude yellow oil (200.0 mg, 100 %) can be used directly for next step without purification. Only for NMR analysis, the product was purified by fast flash chromatography on silica gel (ethyl acetate) then recycling GPC ($CHCl_3$) to give compound **10** as white oily liquid, which solidified slowly.

$^1$H NMR (300 MHz, $CD_2Cl_2$) $\delta$ 7.61 – 7.55 (m, 1H), 7.52 (dd, $J$ = 8.4, 3.8 Hz, 3H), 7.45 – 7.36 (m, 1H), 7.36 – 7.30 (m, 3H), 7.29 (d, $J$ = 1.5 Hz, 1H), 7.26 – 7.21 (m, 1H), 7.21 – 7.14 (m, 2H), 7.12 – 7.05 (m, 1H), 6.97 – 6.85 (m, 3H), 6.82 (d, $J$ = 3.8 Hz, 1H), 6.78 (t, $J$ = 2.7 Hz, 3H), 6.76 – 6.70 (m, 2H);

$^{13}$C NMR (76 MHz, $CD_2Cl_2$) $\delta$ 144.9, 144.8, 142.4, 142.1, 142.1, 142.0, 141.4, 141.2, 140.7, 140.5, 139.1, 139.0, 138.8, 138.6, 138.2, 137.2, 137.0, 132.7, 132.5, 132.3, 132.2, 131.8, 131.1, 130.2, 129.1, 129.1, 128.9, 128.8, 128.7, 128.6, 127.9, 127.8, 127.3, 125.5, 125.4, 125.2, 120.4, 120.1, 119.7, 115.1, 114.8, 114.5, 92.4, 92.1.

Comments: Similar to compound **4**, the $^{13}$C NMR spectrum of compound **10** exhibits more than 20 peaks (Fig. S33). This is mainly caused by the presence of conformational isomers (the different rotation angle between the middle biphenyl group with the iodo-biphenyl-amine group). This result is similar to the previous reported molecules.[6-7] Besides, compound **10** is not very stable under air conditions due to the amino group. During $^{13}$C NMR measurement, there will be some impurities caused by the oxidation reaction. However, these isomers and

impurities will not affect the following reactions. It is highly recommended to use the crude product for the next step directly.

HRMS (MADLI-TOF, m/z): calcd for $C_{36}H_{26}I_2N_2^+$ [M]$^+$ 740.0185, found 740.0179.

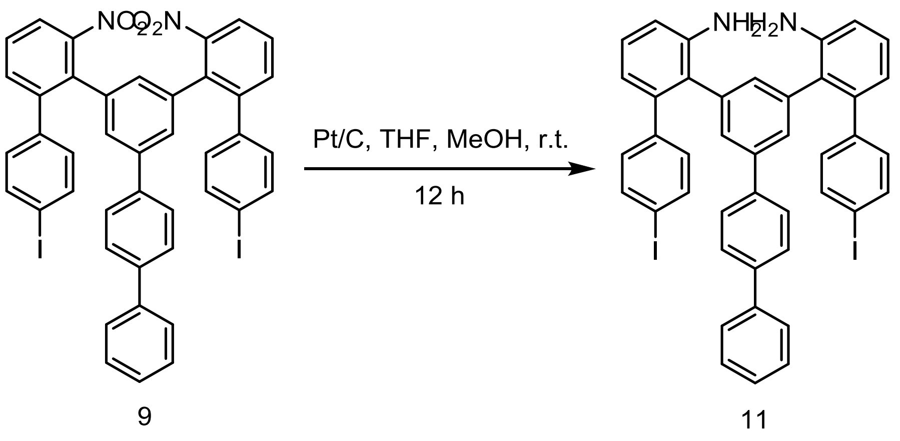

**5''-([1,1'-biphenyl]-4-yl)-4,4''''-diiodo-[1,1':2',1'':3'',1''':2''',1''''-quinquephenyl]-3',6'''-diamine (11).** In a 50 mL one-necked flask, compound **9** (428.3 mg, 0.54 mmol) was charged under the protection of argon. Then 20 mL THF and methanol (1:1) was added into the flask. After Pt/C (84.6 mg, 0.43 mmol) was charged under the protection of argon, the mixture was degassed by $H_2$ for 5 minutes. And the resulting mixture was stirred at room temperature for 12 hours with a $H_2$ balloon. Afterwards the mixture was filtered by celite and the solvent was evaporated in vacuo. The crude yellow oil (440.9 mg, 100 %) can be used directly for next step without purification. Only for NMR analysis, the product was first purified by fast flash chromatography on silica gel (ethyl acetate) then recycling GPC ($CHCl_3$) to give compound **11** as white oily liquid, which solidified slowly.

$^1$H NMR (300 MHz, $CD_2Cl_2$) $\delta$ 7.63 (dd, $J$ = 10.7, 4.5 Hz, 2H), 7.60 – 7.55 (m, 2H), 7.52 (d, $J$ = 8.3 Hz, 2H), 7.49 – 7.45 (m, 1H), 7.45 – 7.39 (m, 2H), 7.39 – 7.34 (m, 2H), 7.32 (d, $J$ = 1.9 Hz, 1H), 7.26 – 7.21 (m, 1H), 7.21 – 7.17 (m, 2H), 7.17 – 7.14 (m, 2H), 7.13 (d, $J$ = 1.4 Hz, 1H), 6.89 (d, $J$ = 8.3 Hz, 2H), 6.84 – 6.80 (m, 1H), 6.80 – 6.77 (m, 3H), 6.74 (dd, $J$ = 7.8, 5.7 Hz, 2H), 3.79 (s, 1H), 3.38 (s, 3H);

$^{13}$C NMR (76 MHz, $CD_2Cl_2$) $\delta$ 144.9, 141.2, 138.9, 138.7, 137.2, 137.1, 132.2, 130.3, 130.2, 129.3, 129.2, 129.1, 129.0, 129.0, 128.8, 128.7, 128.6, 128.5, 127.8, 127.8, 127.7, 127.7, 127.3, 127.2, 120.4, 119.7, 114.9.

Comments: Similar to compound **4**, the $^{13}C$ NMR spectrum of compound **11** exhibits more than 24 peaks (Fig. S36). In addition, the amino signal in $^{1}H$ NMR split into two peaks (Fig. S35). These results are mainly caused by the presence of conformational isomers (the different rotation angle between the middle terphenyl group with the iodo-biphenyl-amine group), which are similar to the previous reported molecules.[6-7] Besides, compound **11** is not very stable under air conditions due to the amino group and exhibit low solubility. During $^{1}H$ and $^{13}C$ NMR measurement, there will be some impurities caused by the oxidation reaction. However, these isomers and impurities will not affect the following reactions. It is highly recommended to use the crude product for the next step directly.

HRMS (MADLI-TOF, m/z): calcd for $C_{42}H_{30}I_2N_2^+$ $[M]^+$ 816.0498, found 816.0502.

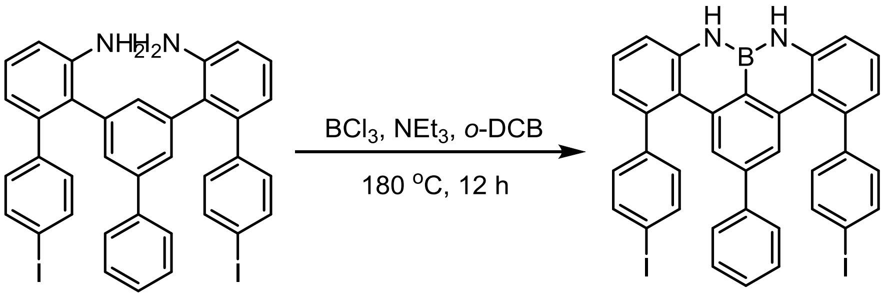


10 M1

**4,13-bis(4-iodophenyl)-2-phenyl-8H,9H-8,9-diaza-8a-borabenzo[fg]tetracene (**M1**).** To a solution of compound **10** (200.0 mg, 0.27 mmol) in *o*-dichlorobenzene (12 mL), triethylamine (80.3 mg, 0.81 mmol) and boron trichloride (1 M in n-hexane, 0.50 mL, 0.50 mmol) were added under argon atmosphere. The reaction mixture was heated to 180 °C for 12 h. After cooling to the room temperature, the solvent was evaporated through reduced pressure distillation at 50 °C (0.01 mbar). The residue was then purified by chromatography on silica gel ($CH_2Cl_2$/iso-hexane = 1/1) then recrystallized two times from $CHCl_3$/MeOH to give compound M1 as white solid (65.0 mg, 32%).

$^{1}H$ NMR (300 MHz, $C_2D_2Cl_4$) $\delta$ 7.82 (d, J = 1.7 Hz, 2H), 7.79 (d, J = 2.3 Hz, 2H), 7.41 (d, J = 3.9 Hz, 2H), 7.37 (d, J = 7.8 Hz, 2H), 7.31 (t, J = 7.7 Hz, 2H), 7.24 (d, J = 7.3 Hz, 1H), 7.19 (d, J = 1.8 Hz, 2H), 7.16 (s, 2H), 7.06 (d, J = 7.7 Hz, 2H), 6.88 (d, J = 7.1 Hz, 2H), 6.61 – 6.56 (m, 2H), 6.43 (s, 2H);

$^{11}B$ NMR (96 MHz, $C_2D_2Cl_4$) $\delta$ 27.20;

$^{13}C$ NMR (76 MHz, $C_2D_2Cl_4$) $\delta$ 145.6, 142.4, 141.2, 140.7, 138.7, 131.5, 129.5, 128.0, 127.8, 127.1, 125.0, 124.8, 118.7, 100.0, 92.8

Comments: The room temperature $^{13}C$ NMR spectrum of M1 doesn't show 19 peaks due to the bad solubility (Fig. S39). Besides, there are some very small peaks appeared in the aromatic area from room temperature $^{1}H$ NMR spectrum of M1 (Fig. S38). Based on the previous reported molecules,[6-7] we thought it may cause by the presence of conformational isomers (the different rotation angle between the middle benzene group with the iodo-benzene group). We optimized the structure of different conformational isomers for M1 by DFT calculation and it clearly shows the energy difference. Anyway, these isomers will not affect the on-surface reaction.

HRMS (MADLI-TOF, m/z): calcd for $C_{36}H_{23}BI_2N_2^+$ $[M]^+$ 748.0044, found 748.0037.

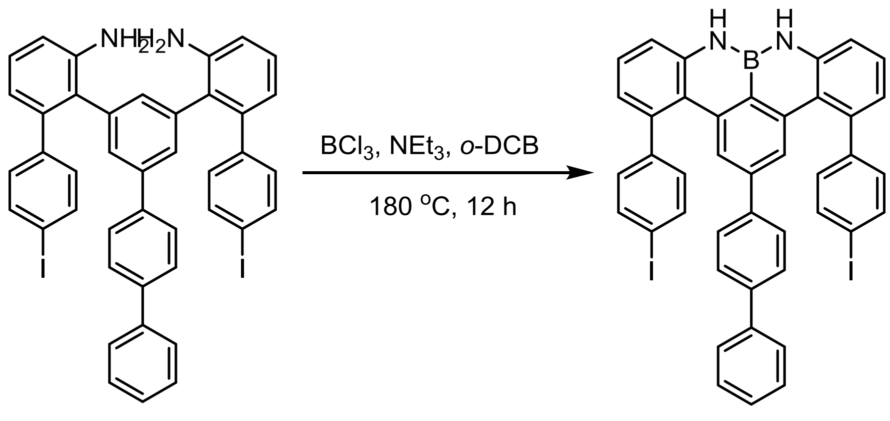


**2-([1,1'-biphenyl]-4-yl)-4,13-bis(4-iodophenyl)-8H,9H-8,9-diaza-8a-borabenzo[fg]tetra-cene (**M2**).** To a solution of compound **11** (440.9 mg, 0.54 mmol) in *o*-dichlorobenzene (20 mL), triethylamine (185.0 mg, 1.83 mmol) and boron trichloride (1 M in n-hexane, 0.92 mL, 0.92 mmol) were added under argon atmosphere. The reaction mixture was heated to 180 °C for 12 h. After cooling to the room temperature, the solvent was evaporated through reduced pressure distillation at 50 °C (0.01 mbar). The residue was then purified by chromatography on silica gel ($CH_2Cl_2$/iso-hexane = 1/1) then recrystallized two times from $CHCl_3$/MeOH to give compound M2 as white powder (146.9 mg, 33%).

$^{1}H$ NMR (300 MHz, $C_2D_2Cl_4$) $\delta$ 7.87 (d, J = 1.7 Hz, 2H), 7.84 (d, J = 1.8 Hz, 2H), 7.69 (dd, J = 16.6, 5.5 Hz, 2H), 7.65 – 7.58 (m, 2H), 7.53 (d, J = 6.7 Hz, 2H), 7.48 (d, J = 6.6 Hz, 2H), 7.45

– 7.37 (m, 1H), 7.33 (t, J = 7.6 Hz, 2H), 7.24 (s, 2H), 7.22 (d, J = 0.9 Hz, 2H), 7.08 (d, J = 8.2 Hz, 2H), 6.94 (d, J = 7.3 Hz, 2H), 6.77 (d, J = 8.2 Hz, 2H), 6.33 (s, 2H);

$^{13}C$ NMR (76 MHz, $C_2D_2Cl_4$) $\delta$ 145.8, 142.5, 141.4, 138.6, 131.5, 129.3, 129.1, 127.8, 127.8, 127.7, 127.6, 127.6, 127.5, 127.4, 127.4, 125.0, 124.8, 118.5, 99.9, 92.5.

Comments: The high temperature (393K) $^{13}C$ NMR spectrum of M2 does not show 23 peaks due to the very bad solubility (Fig. S47). In contrast to M1, there is no obvious isomer's signal appeared in the aromatic area from high temperature $^{1}H$ NMR spectrum of M2 (Fig. S46), which may cause by the higher energy difference between different isomers and the bad solubility of M2. Anyway, for the on-surface reaction these is no affection.

HRMS (MADLI-TOF, m/z): calcd for $C_{42}H_{27}BI_2N_2^+$ $[M]^+$ 824.0357, found 824.0353.

## 3. STM results

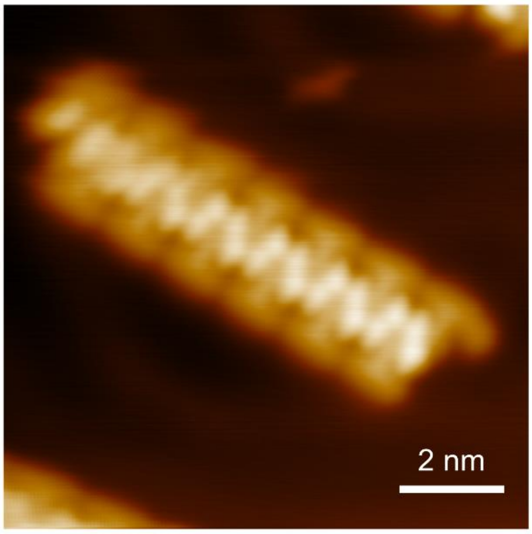


**Figure S1**: STM image of M1 assembled structures as sublimed on Au(111) (V = −300 mV, I = 110 pA). The bright protrusions along the middle region of the assembly are benzene rings D which have rotated due to steric hindrance. The less bright peripheral parts along the assembly are the NBN direction.

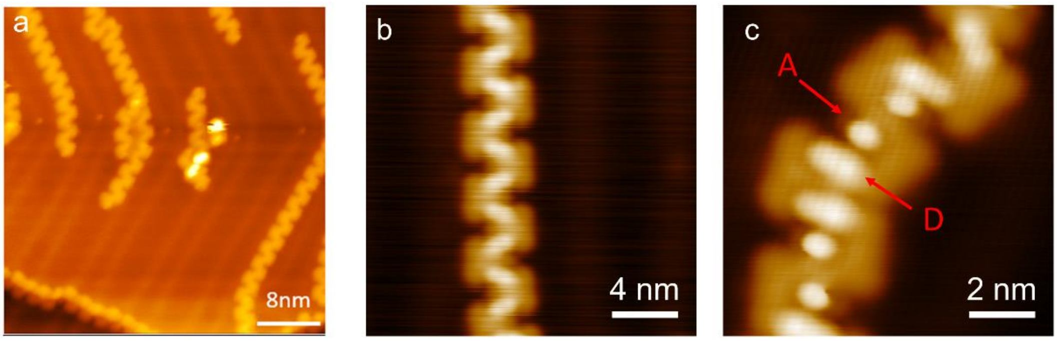


**Figure S2**: (a) STM image of *poly*-***1*** on Au(111); (b) High resolution STM image of *poly*-***1*** on Au(111) acquired with a CO-functionalized tip. The bright protrusions are rotated benzene rings D or A. A straight segment of *poly*-***1*** where all the benzene rings A rotate away from the surface (V = −500 mV, I = 100 pA); (c) A curved segment of *poly*-***1*** where benzene rings D and A randomly rotate away from the surface (V = −650 mV, I = 40 pA).

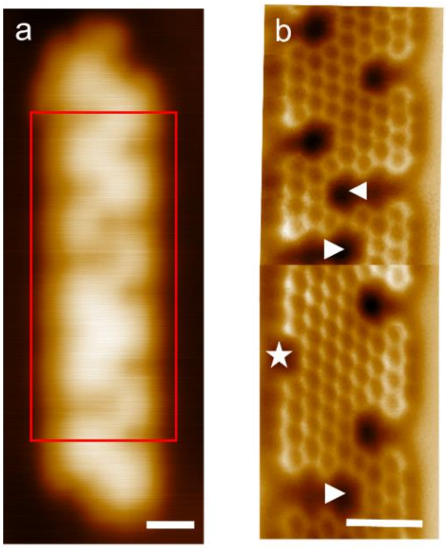


**Figure S3**: (a) STM image of NBN-ZGNR1 with defects (V = −1 V, I = 30 pA). (b) AFM image of the red rectangle area in (a). The triangles mark units where benzene rings A are missing. The star marks a unit where an additional benzene ring attached to the benzene ring A. Scale bars: 1 nm.

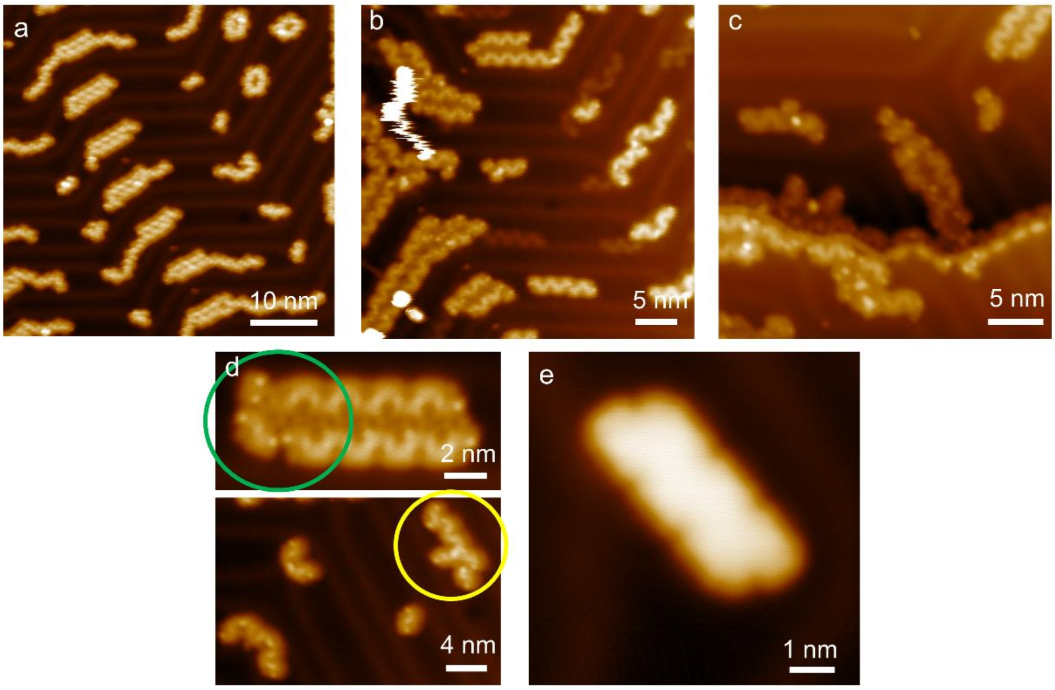


**Figure S4**: (a) STM image of M2 assembled structures as sublimed on Au(111) (V = −1 V, I = 30 pA). (b-d) STM image of *poly-**2*** after M2 annealed at 270 °C on Au(111) (V = −1 V, I = 10 pA). (e) Zoom-in STM image of NBN-ZGNR2 (V = −1 V, I = 30 pA).

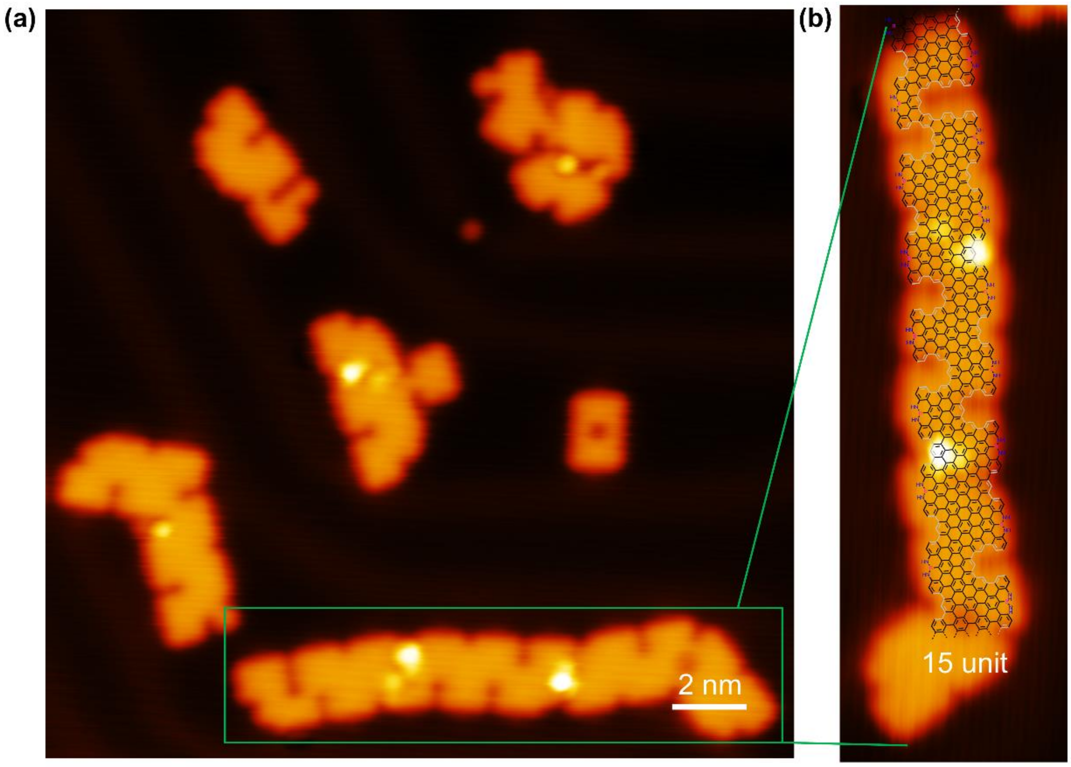


**Figure S5**: (a) STM image of NBN-ZGNR2 with a length of 12.3 nm. (b) The locally enlarged image of Figure S5a, there are 15 repeating units (about 12.3 nm) in this ribbon, which bears defects of missing benzene rings and some contaminations.

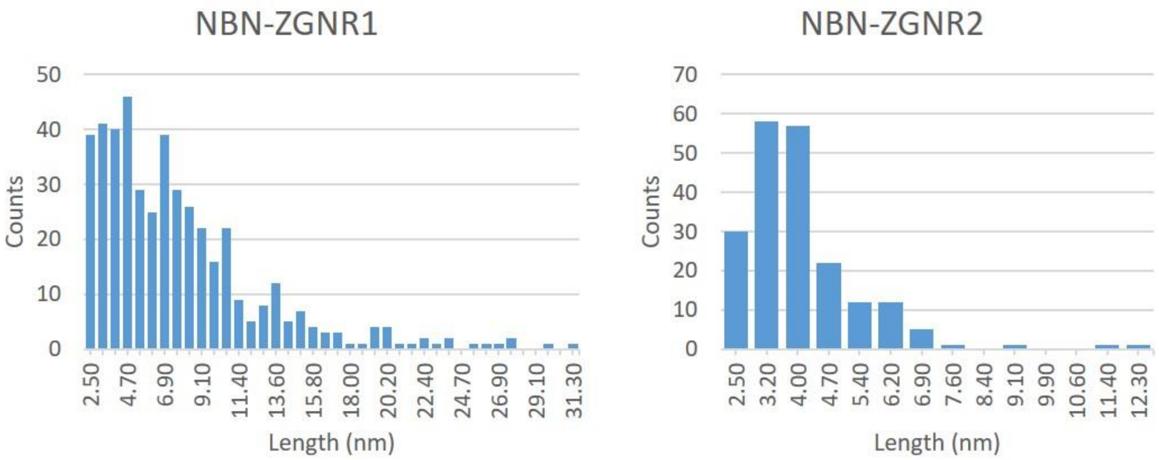


**Figure S6**: The statistical length distribution of NBN-ZGNR1 and NBN-ZGNR2. Dimers are excluded in the analysis. The shortest ribbon consists of 3 monomers, which equals to 2.5 nm.

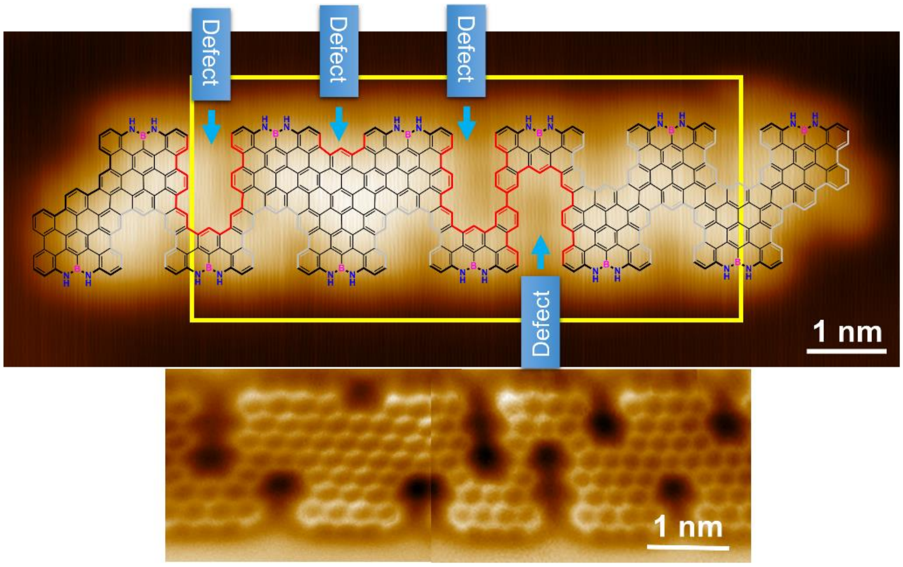


**Figure S7**: Defects in the longer NBN-ZGNR1.

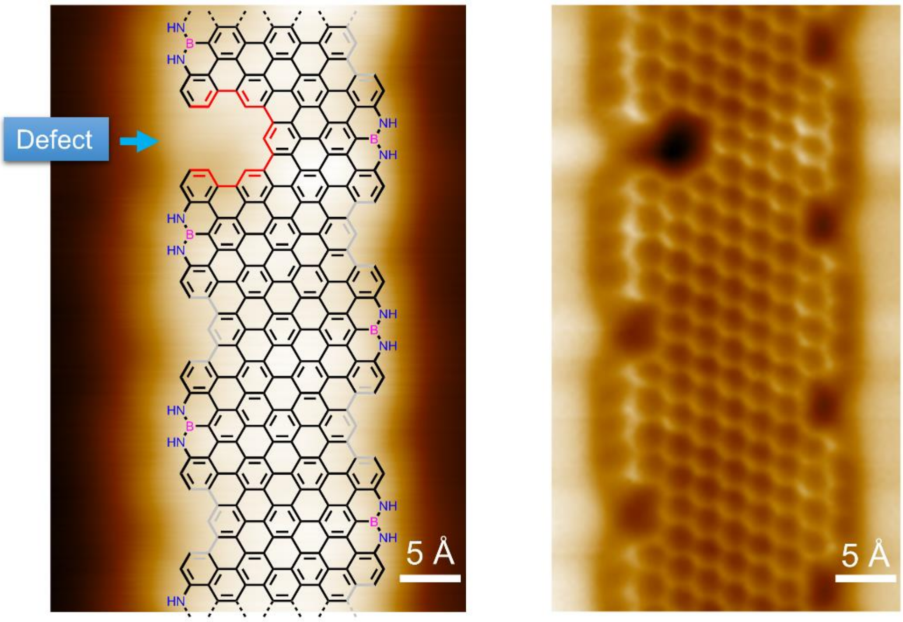


**Figure S8**: Defects in the longer NBN-ZGNR2.

## 4. Assignment of zigzag edge proportion

The assignment of zigzag edge proportion is based on the reported literature.[8] The definition of zigzag and armchair edges is defined by their bond ratio as pictured in Figure S9a,b. By this definition, 32 zigzag and 56 armchair bonds are found in NBN-ZGNR1 as represented in Figure S9c. Accordingly, the zigzag edge proportions in NBN-ZGNR1 is 36 %. Similar to NBN-ZGNR1, 32 zigzag bonds are found in NBN-ZGNR2 while only 24 armchair bonds are found (Figure S9d). Thus it leads to a rising zigzag edge proportion for NBN-ZGNR2 to 57 %.

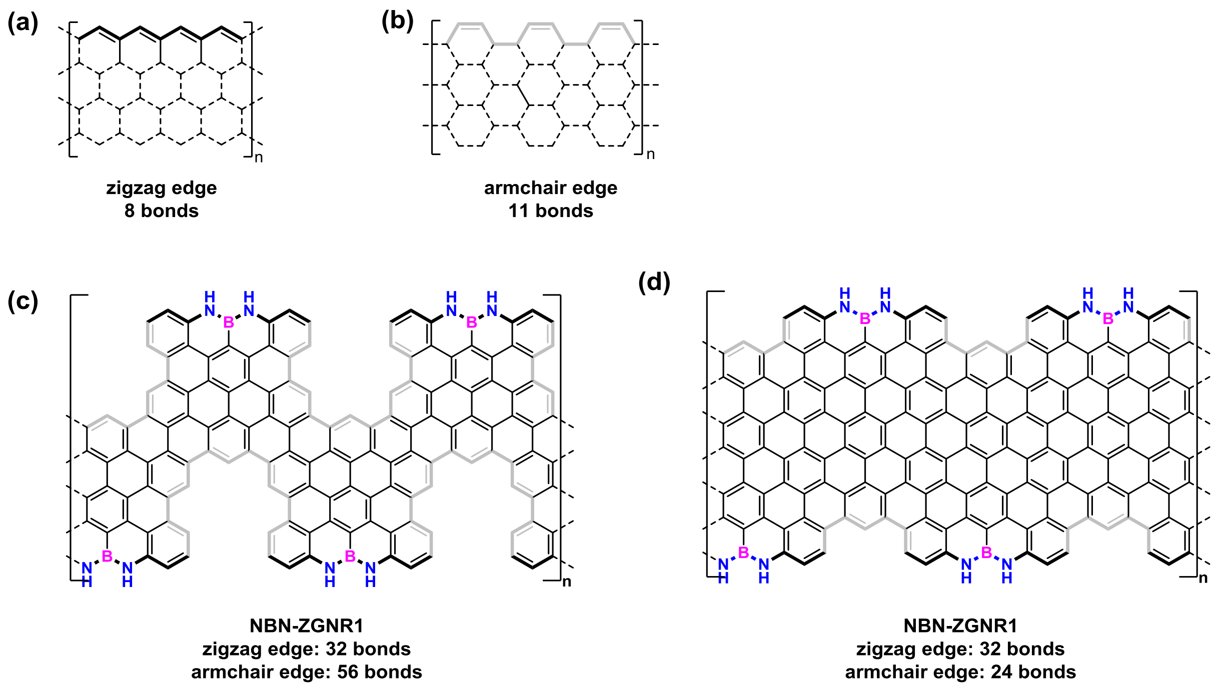


**Figure S9**: (a-b) Explanation of zigzag and armchair edge structure. (c-d) Assignment of zigzag edge carbon bonds in NBN-ZGNR1 and NBN-ZGNR2.

## 5. Details of the theoretical calculations

Density functional theory calculations were performed using the Perdew–Burke–Ernzerhof (PBE) generalized gradient approximation[9]. The Vienna ab initio simulation package (VASP)[10-11] was used to perform the structural relaxations and electronic-structure calculations. The wave functions were expanded using a planewave basis set with an energy cutoff of 500 eV. The vacuum was set to 18 Å to avoid interactions between neighboring nanoribbons. The structures were relaxed until the residual forces were smaller than 0.01 eV/Å. A Γ-centered 6 × 1 × 1 k-point sampling in the 1st Brillouin zone (BZ) was used during optimization of the graphene nanoribbon. The band structure was calculated along high symmetry directions in the Brillouin zone.

# 6. NMR spectra

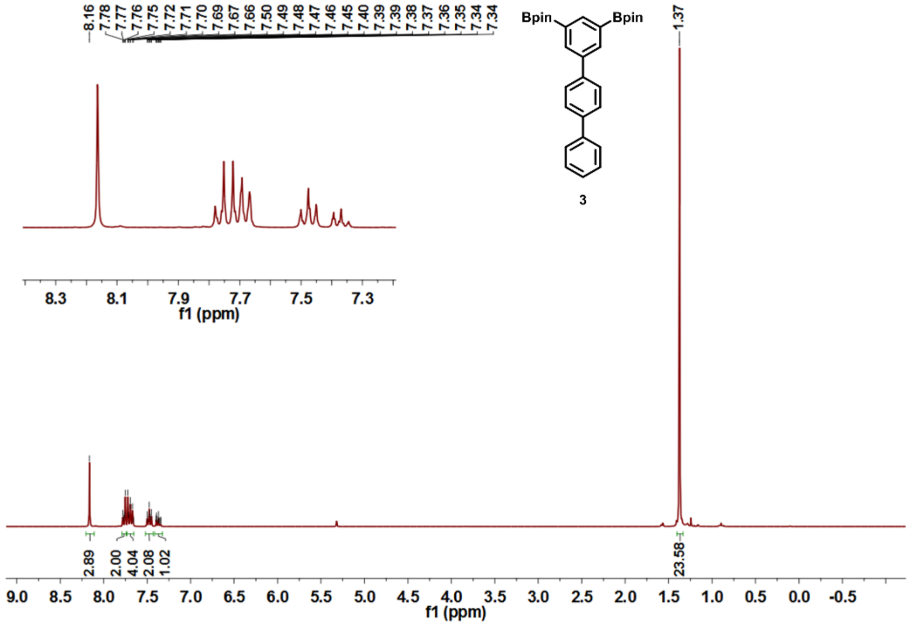


**Figure S10**: 300 MHz $^{1}$H NMR spectrum of compound **3** in $CD_2Cl_2$ at room temperature.

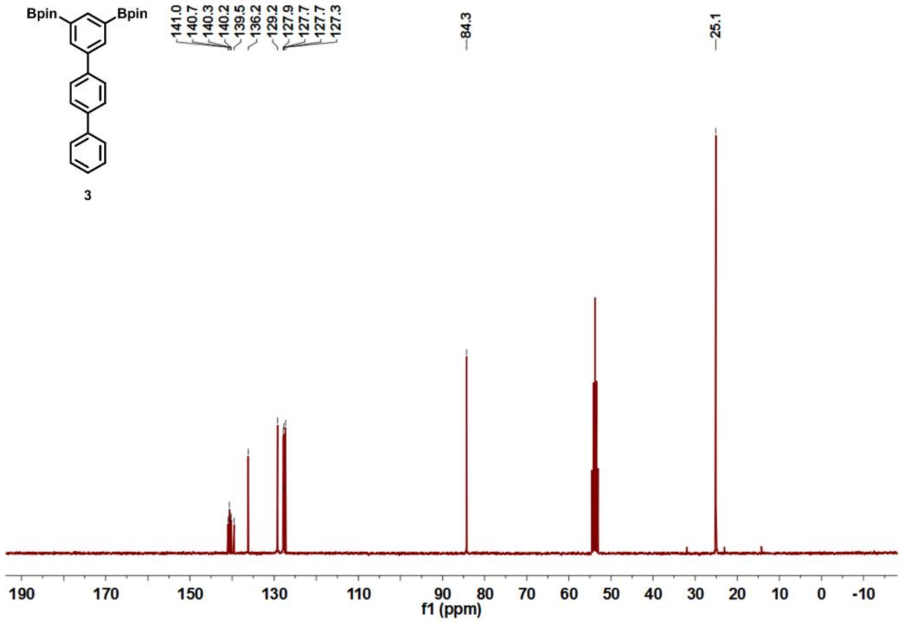


**Figure S11**: 76 MHz $^{13}$C NMR spectrum of compound **3** in $CD_2Cl_2$ at room temperature.

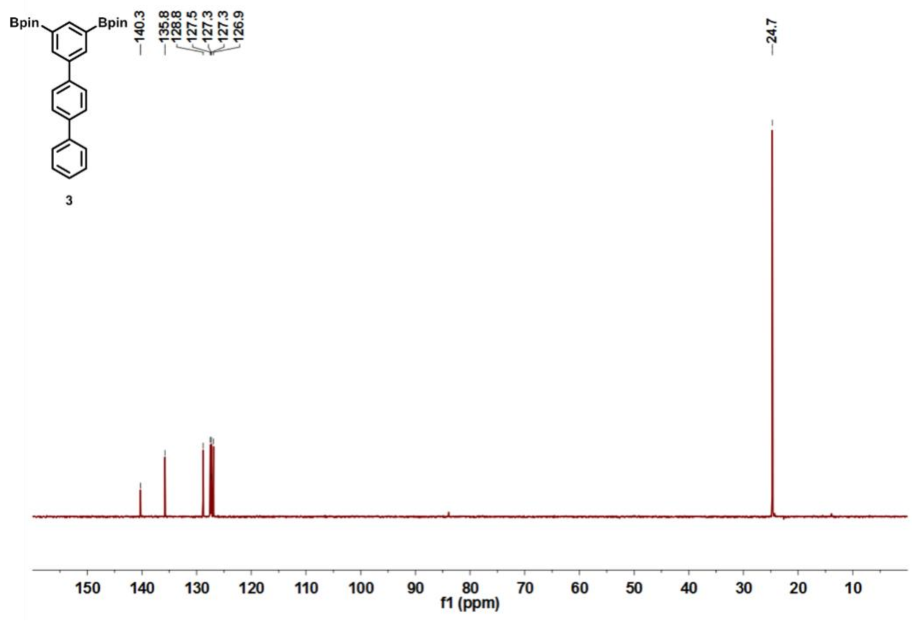


**Figure S12**: 76 MHz $^{13}C$-DEPT-135 NMR spectrum of compound **3** in $CD_2Cl_2$ at room temperature.

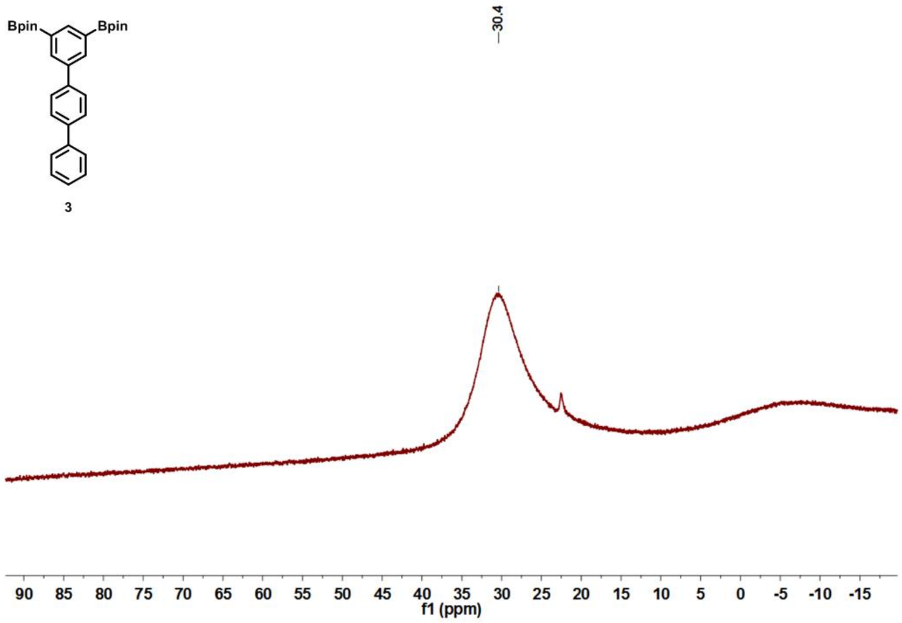


**Figure S13**: 96 MHz $^{11}B$ NMR spectrum of compound **3** in $CD_2Cl_2$ at room temperature.

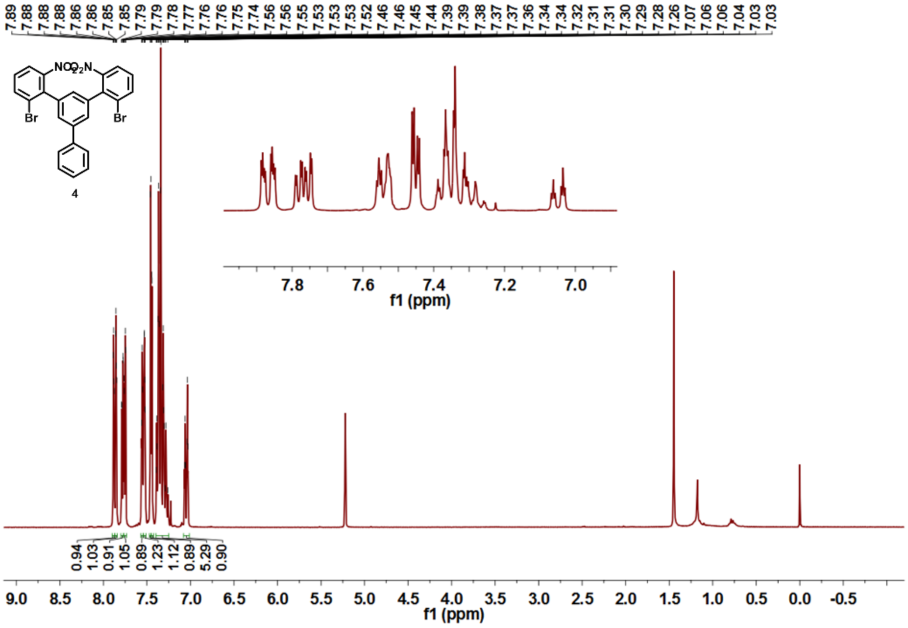


**Figure S14**: 300 MHz $^{1}H$ NMR spectrum of compound **4** in $CD_2Cl_2$ at room temperature.

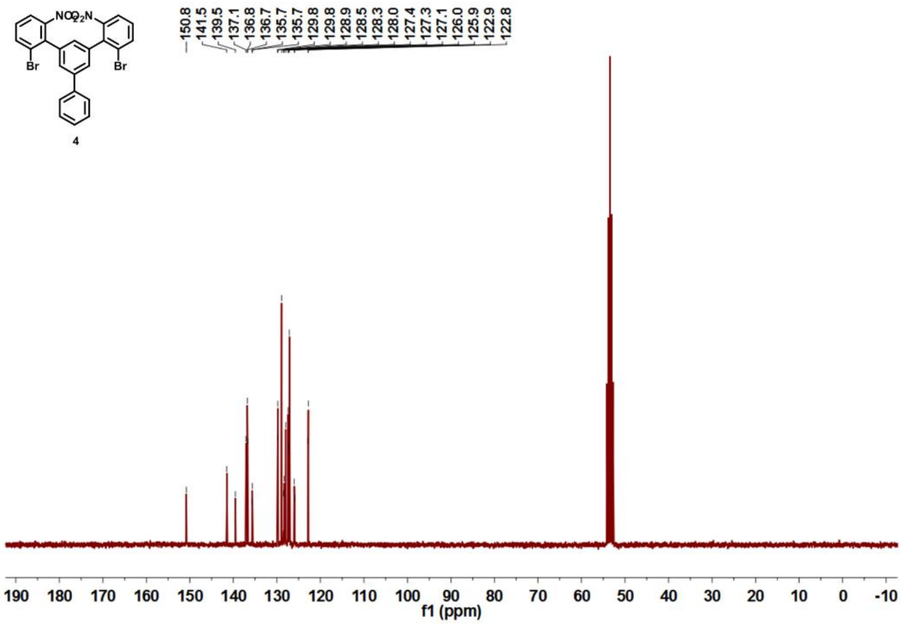


**Figure S15**: 76 MHz $^{13}C$ NMR spectrum of compound **4** in $CD_2Cl_2$ at room temperature.

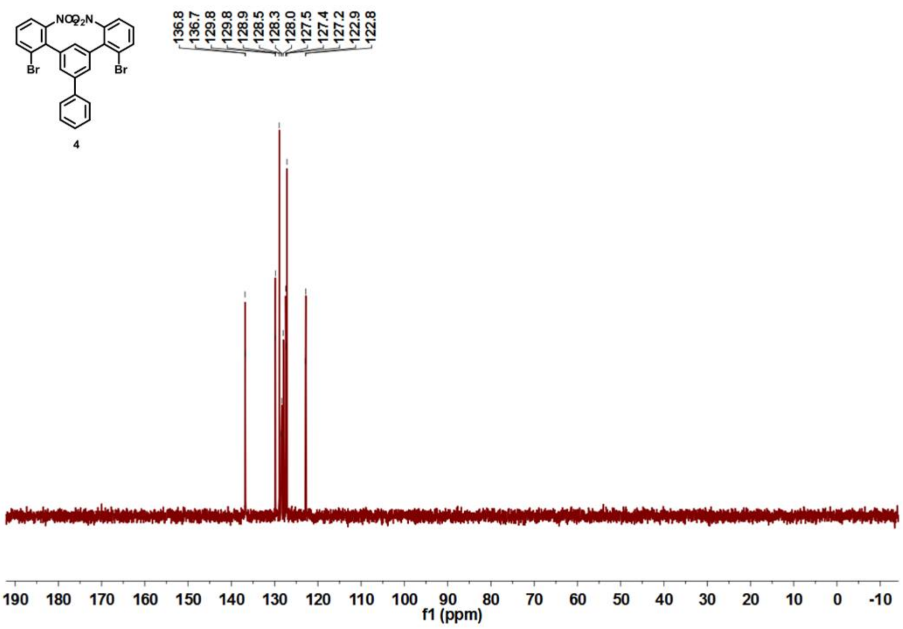


**Figure S16**: 76 MHz $^{13}$C-DEPT-135 NMR spectrum of compound **4** in $CD_2Cl_2$ at room temperature.

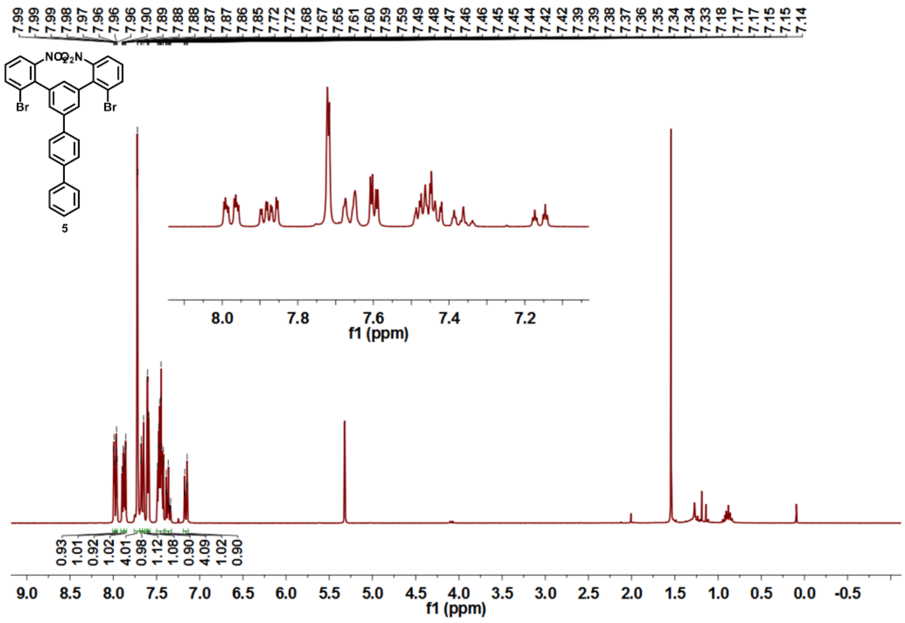


**Figure S17**: 300 MHz $^{1}$H NMR spectrum of compound **5** in $CD_2Cl_2$ at room temperature.

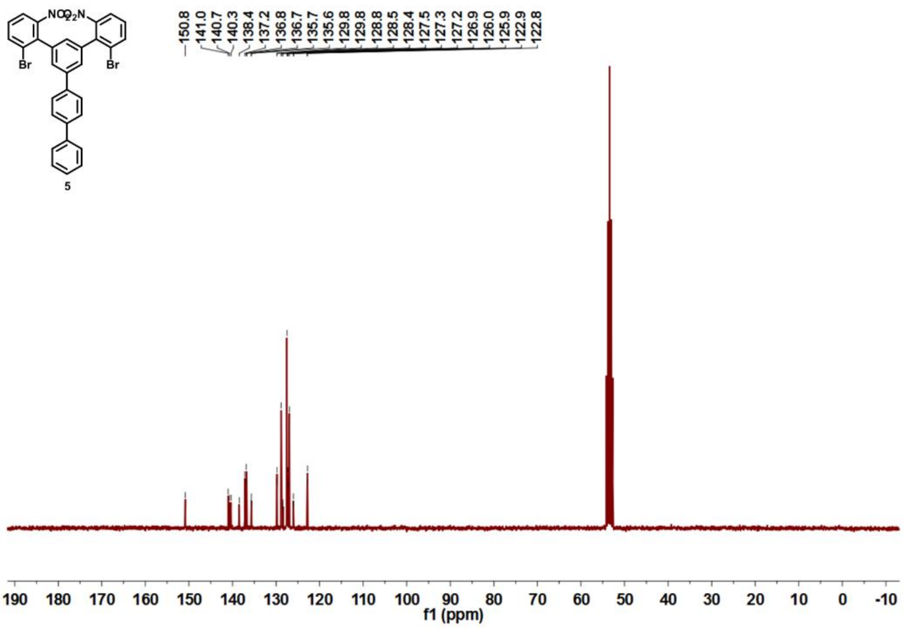


**Figure S18**: 76 MHz $^{13}C$ NMR spectrum of compound **5** in $CD_2Cl_2$ at room temperature.

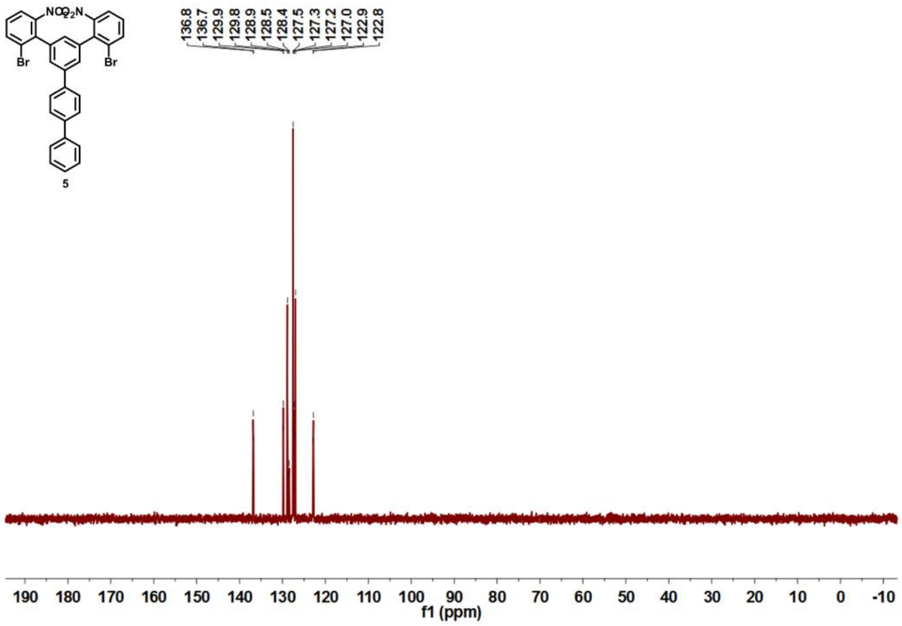


**Figure S19**: 76 MHz $^{13}C$-DEPT-135 NMR spectrum of compound **5** in $CD_2Cl_2$ at room temperature.

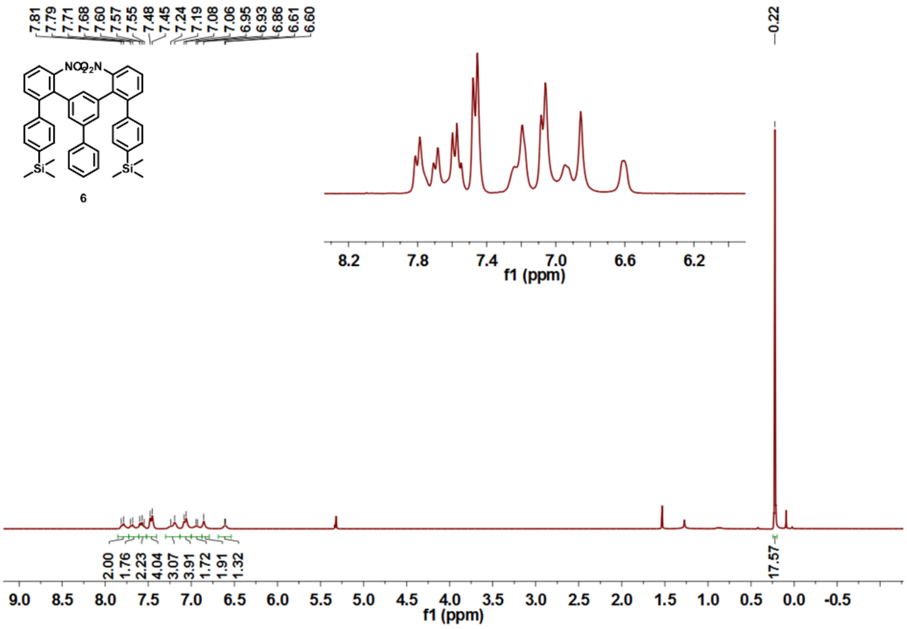

**Figure S20**: 300 MHz $^{1}$H NMR spectrum of compound **6** in $CD_2Cl_2$ at room temperature.

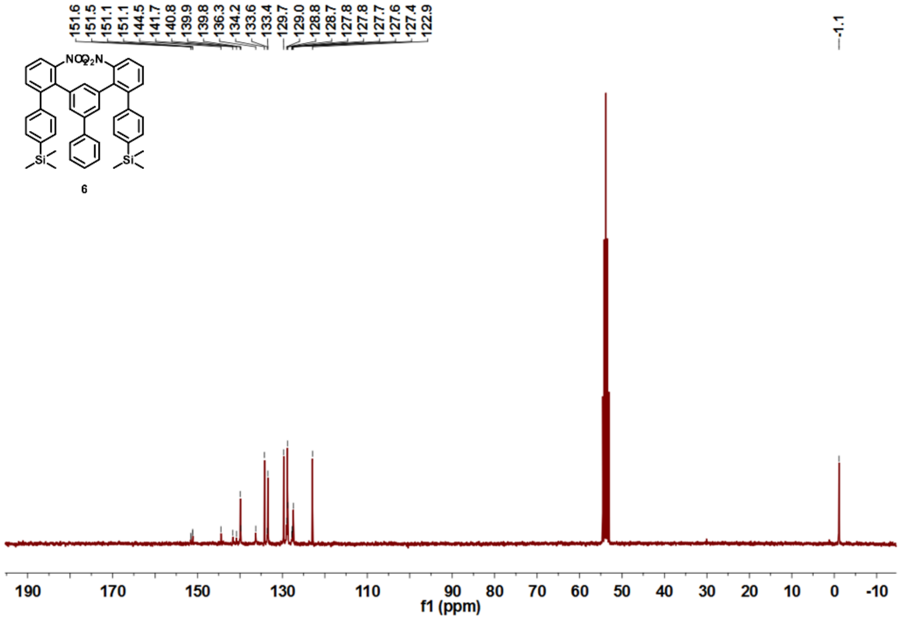

**Figure S21**: 76 MHz $^{13}$C NMR spectrum of compound **6** in $CD_2Cl_2$ at room temperature.

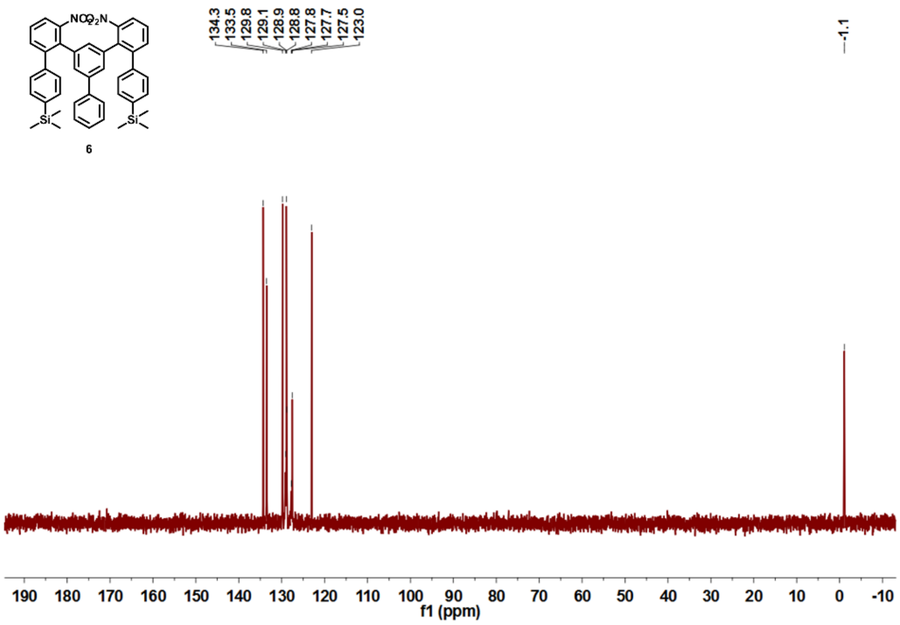


**Figure S22**: 76 MHz $^{13}$C-DEPT-135 NMR spectrum of compound **6** in $CD_2Cl_2$ at room temperature.

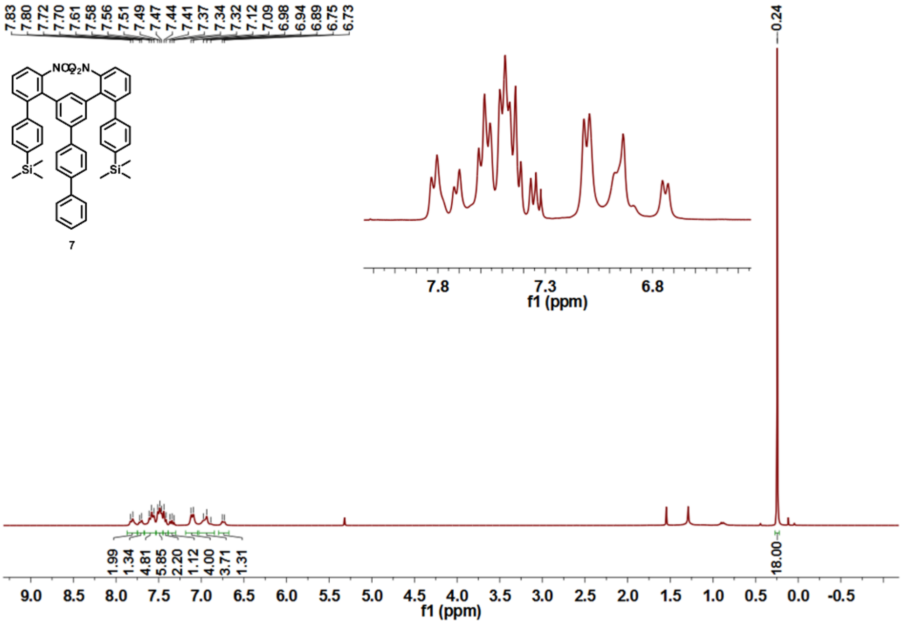


**Figure S23**: 300 MHz $^{1}$H NMR spectrum of compound **7** in $CD_2Cl_2$ at room temperature.

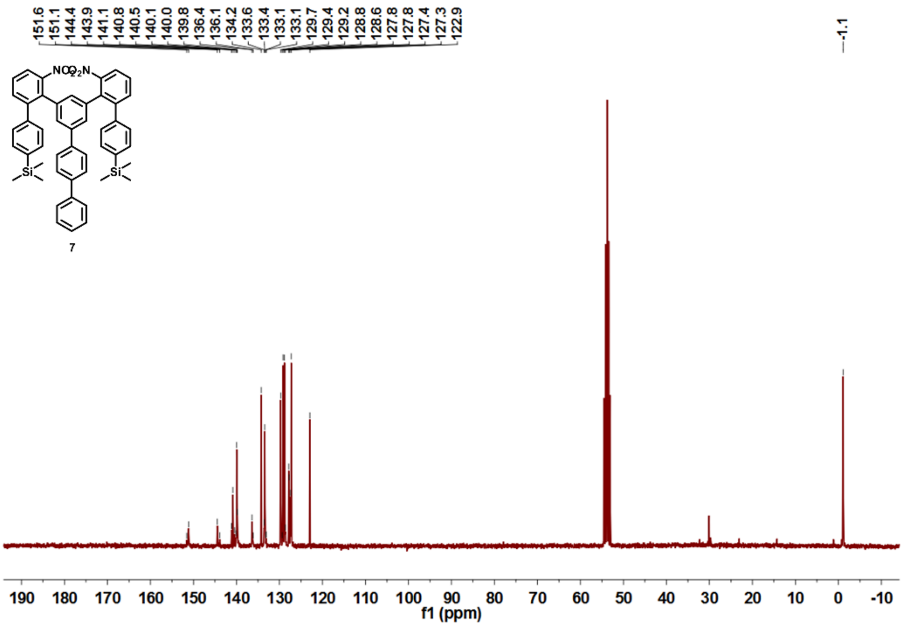


**Figure S24**: 76 MHz $^{13}$C NMR spectrum of compound **7** in $CD_2Cl_2$ at room temperature.

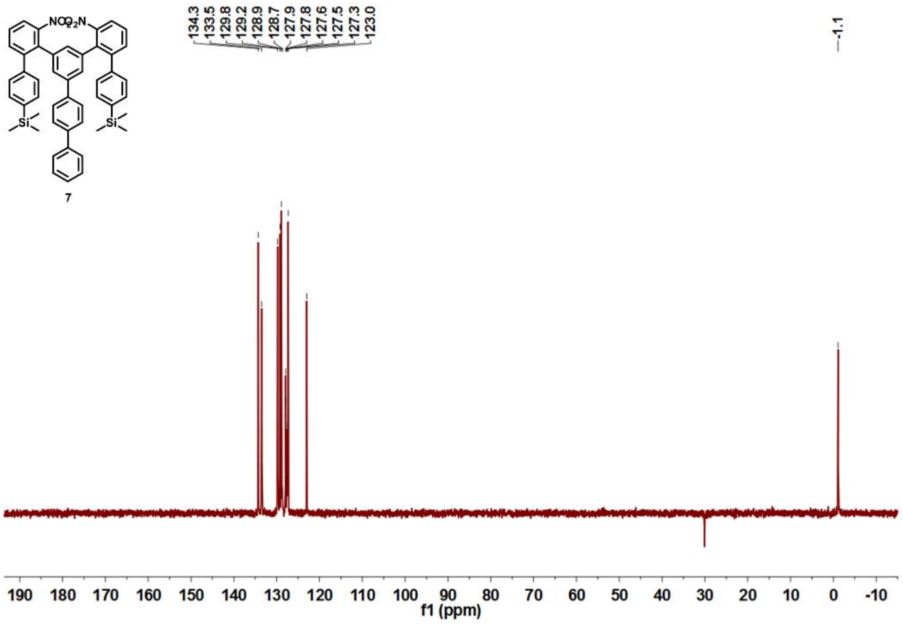


**Figure S25**: 76 MHz $^{13}$C-DEPT-135 NMR spectrum of compound **7** in $CD_2Cl_2$ at room temperature.

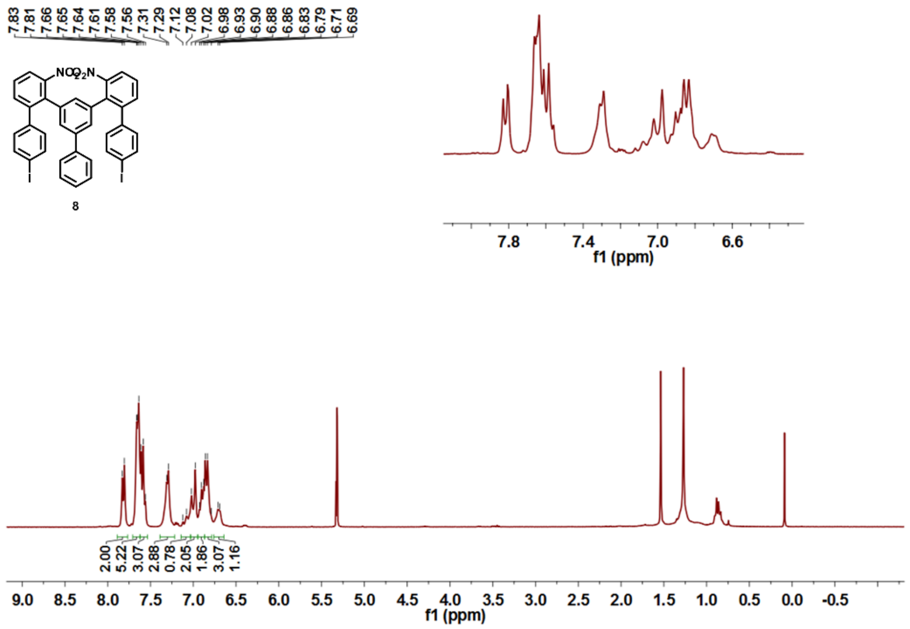

**Figure S26**: 300 MHz $^{1}$H NMR spectrum of compound **8** in $CD_2Cl_2$ at room temperature.

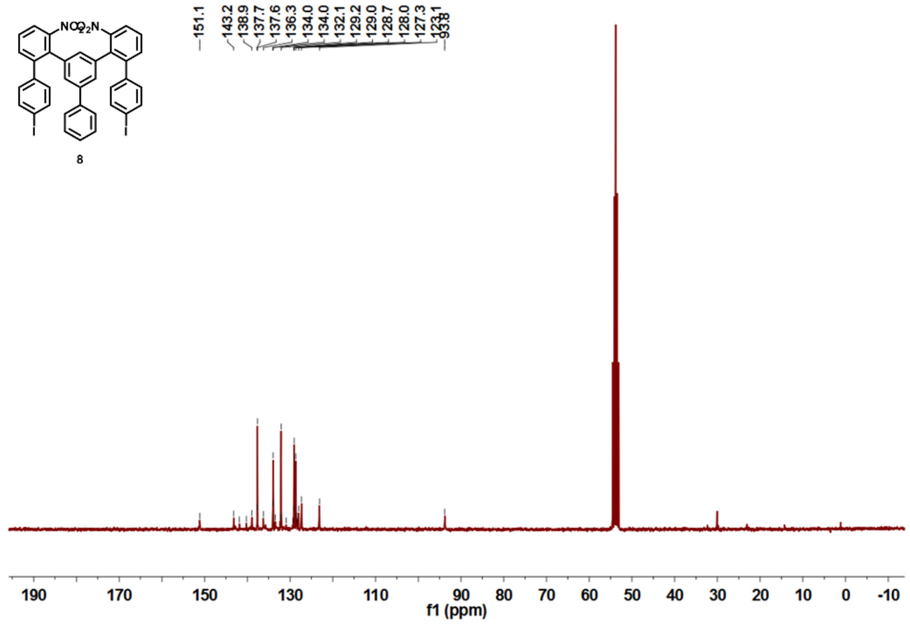

**Figure S27**: 76 MHz $^{13}$C NMR spectrum of compound **8** in $CD_2Cl_2$ at room temperature.

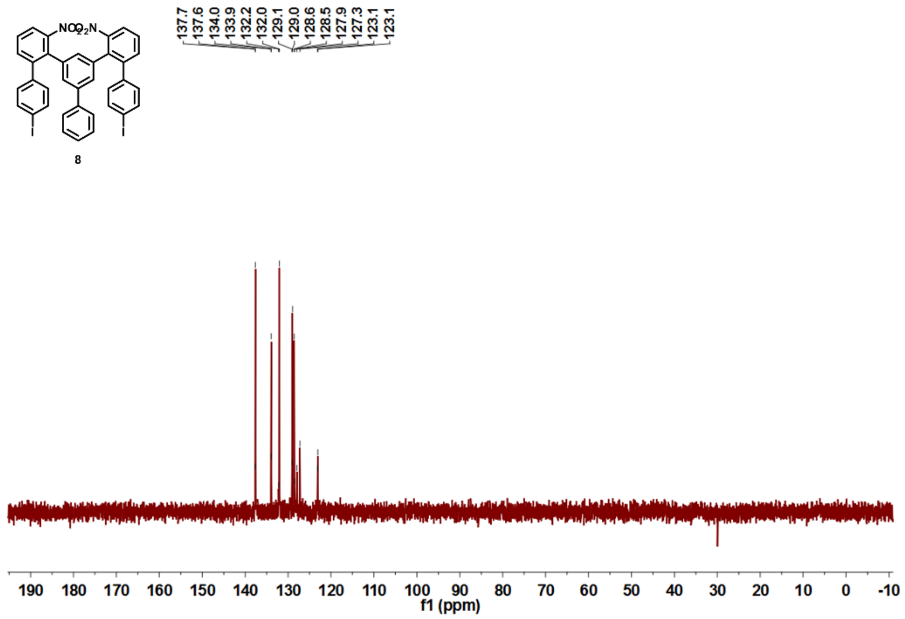

**Figure S28**: 76 MHz $^{13}$C-DEPT-135 NMR spectrum of compound **8** in $CD_2Cl_2$ at room temperature.

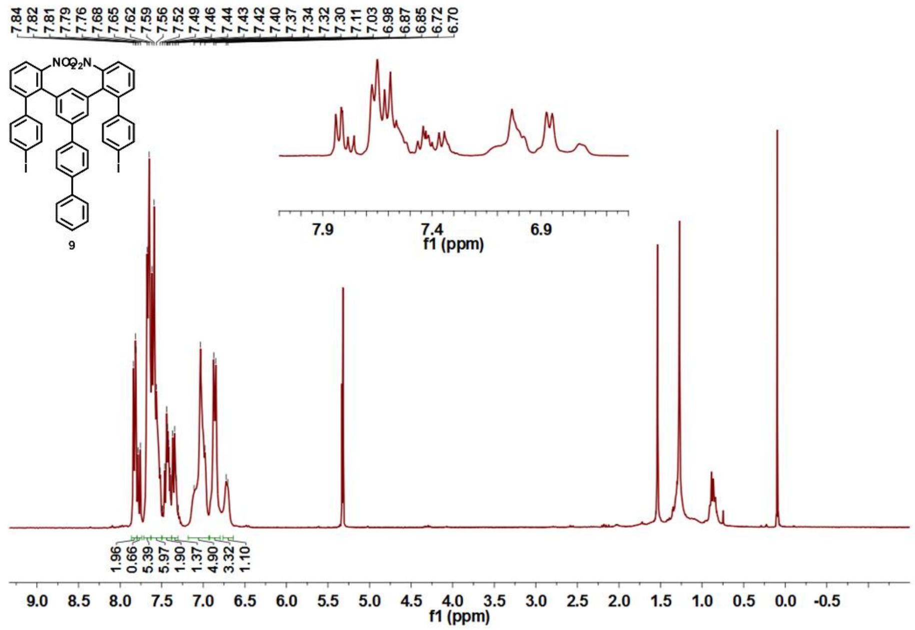

**Figure S29**: 300 MHz $^{1}$H NMR spectrum of compound **9** in $CD_2Cl_2$ at room temperature.

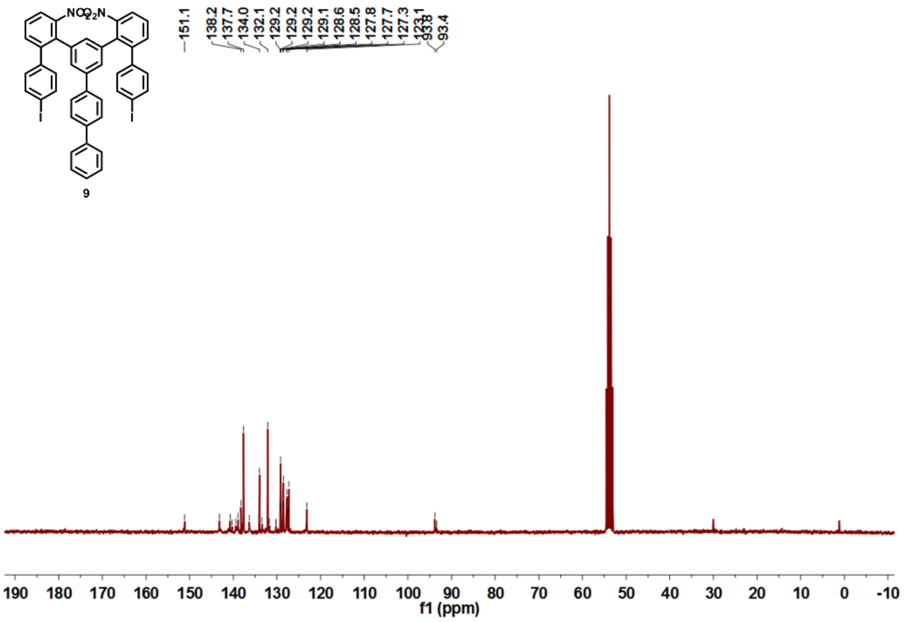


**Figure S30**: 76 MHz $^{13}C$ NMR spectrum of compound **9** in $CD_2Cl_2$ at room temperature.

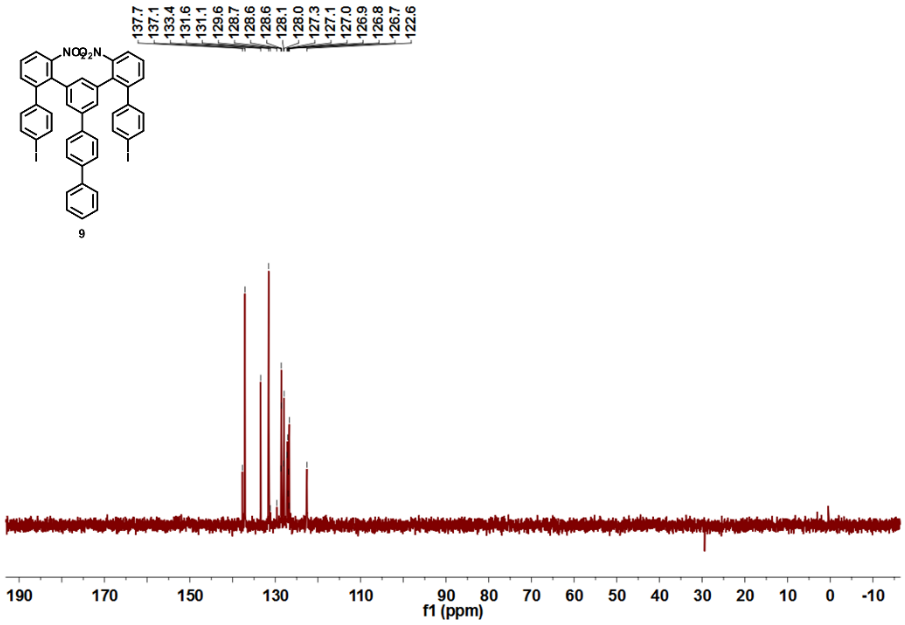


**Figure S31**: 76 MHz $^{13}C$-DEPT-135 NMR spectrum of compound **9** in $CD_2Cl_2$ at room temperature.

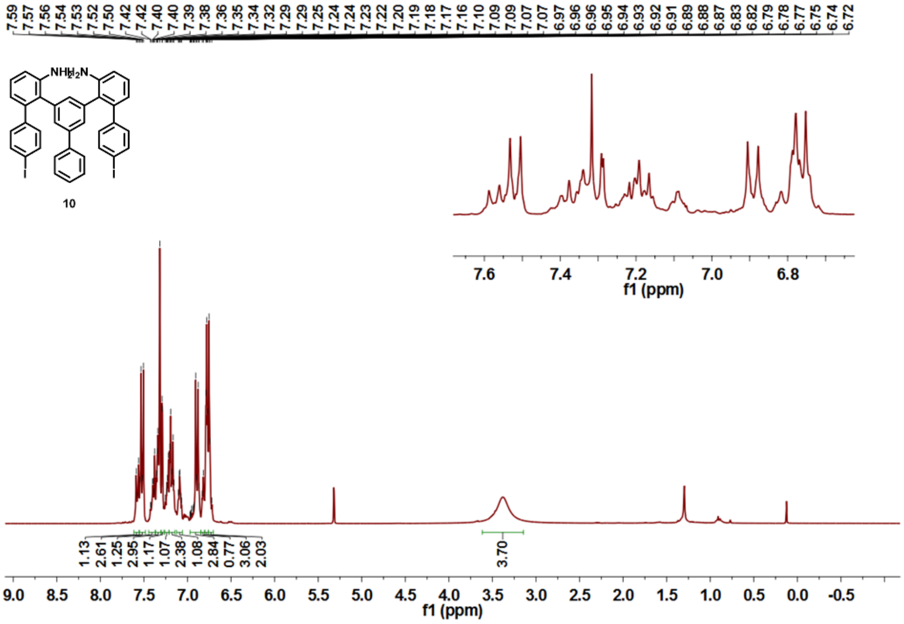


**Figure S32**: 300 MHz $^{1}$H NMR spectrum of compound **10** in $CD_2Cl_2$ at room temperature.

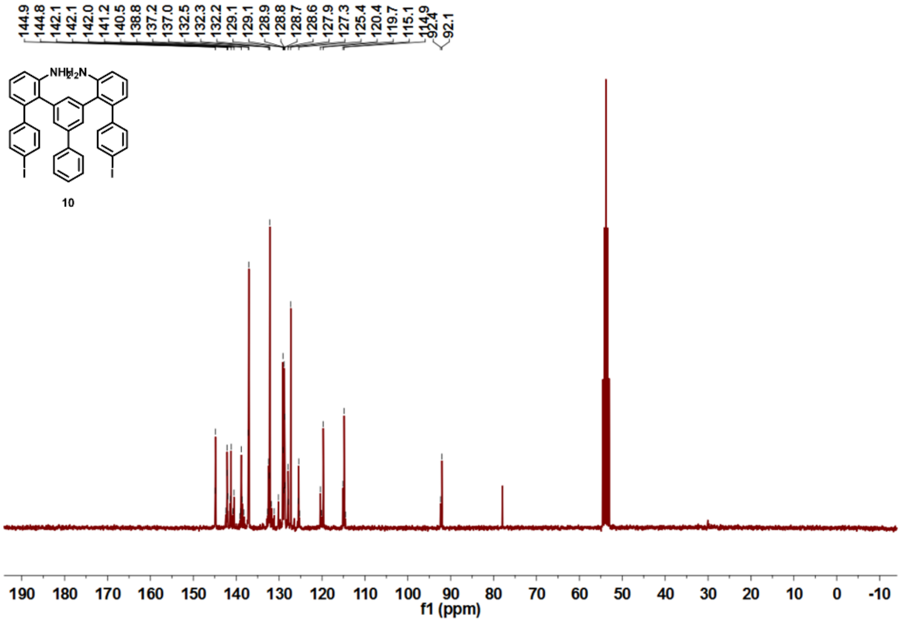


**Figure S33**: 76 MHz $^{13}$C NMR spectrum of compound **10** in $CD_2Cl_2$ at room temperature.

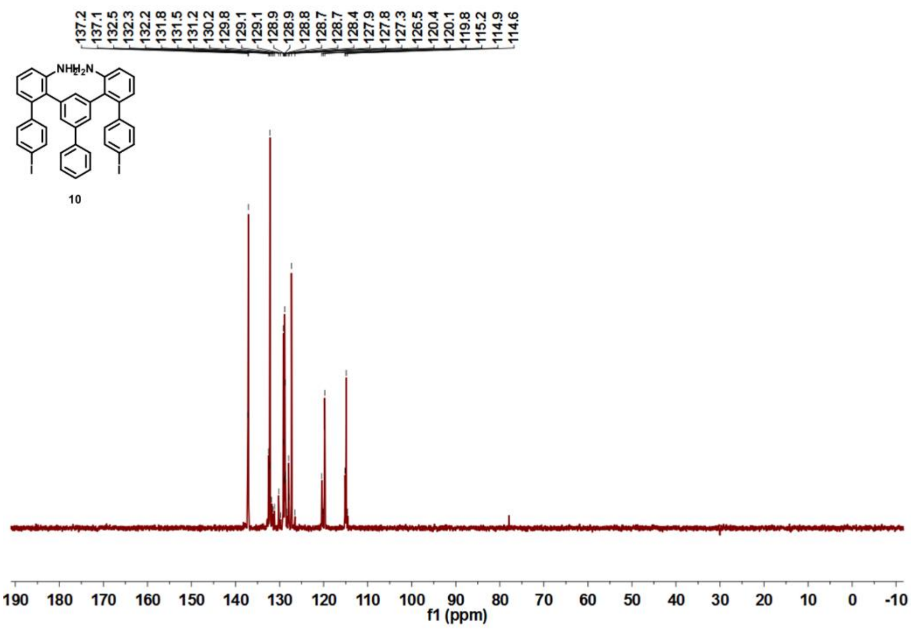


**Figure S34**: 76 MHz $^{13}$C-DEPT-135 NMR spectrum of compound **10** in $CD_2Cl_2$ at room temperature.

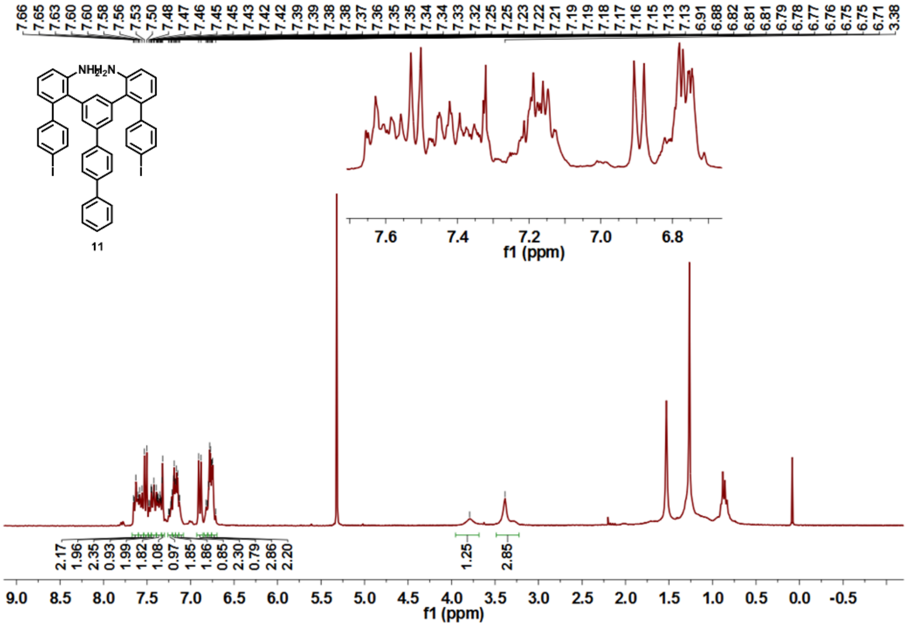


**Figure S35**: 300 MHz $^{1}$H NMR spectrum of compound **11** in $CD_2Cl_2$ at room temperature.

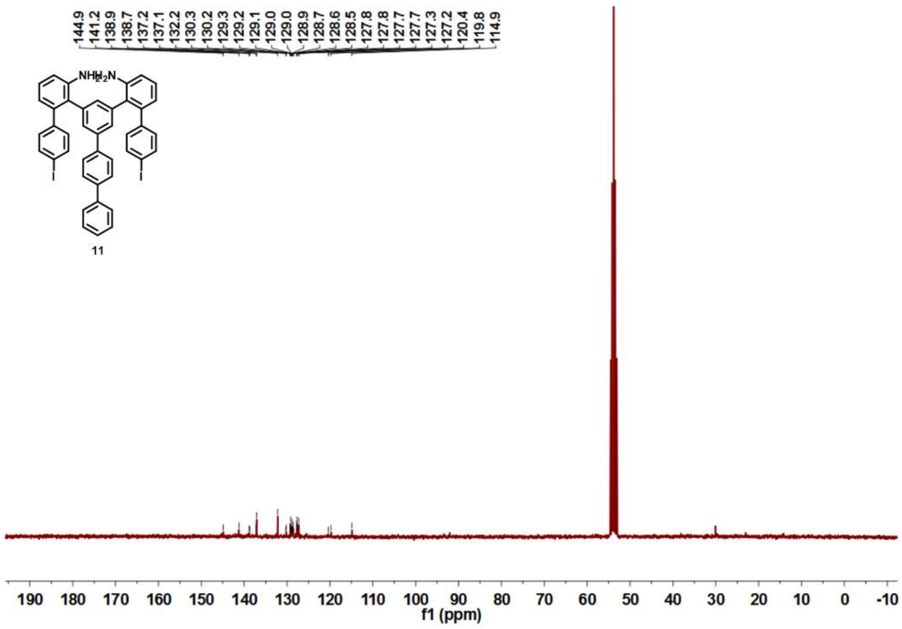

**Figure S36**: 76 MHz $^{13}C$ NMR spectrum of compound **11** in $CD_2Cl_2$ at room temperature.

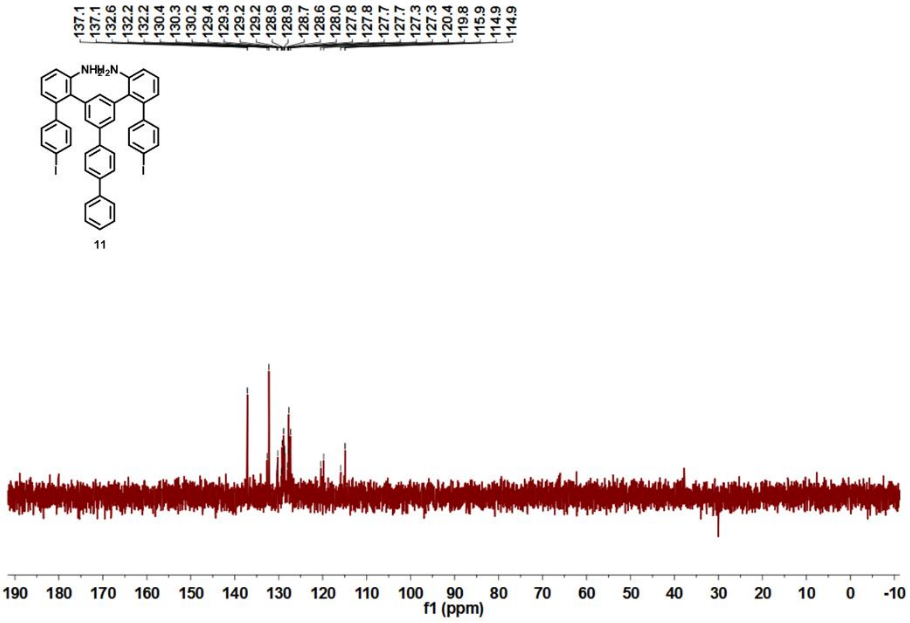

**Figure S37**: 76 MHz $^{13}C$-DEPT-135 NMR spectrum of compound **11** in $CD_2Cl_2$ at room temperature.

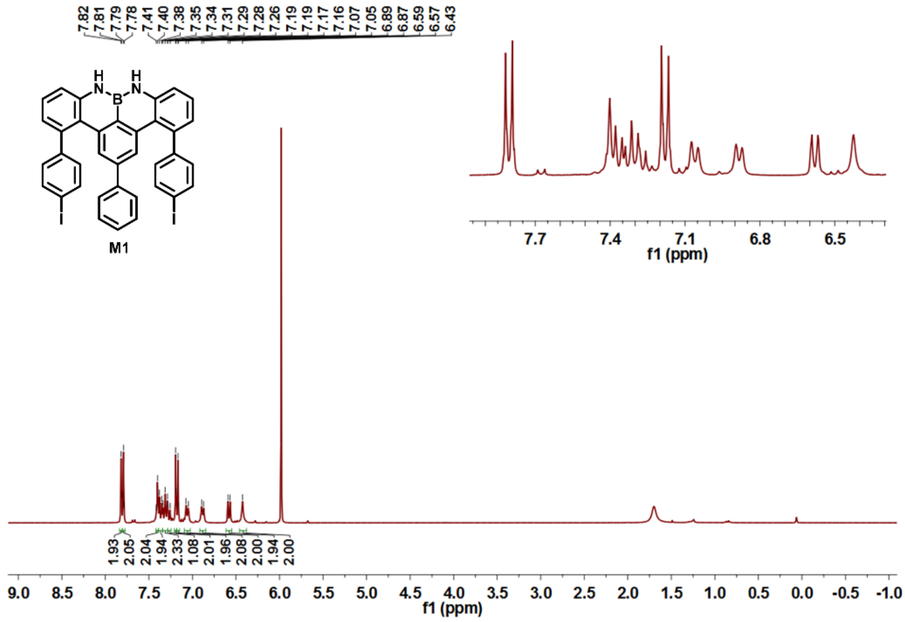

**Figure S38**: 300 MHz $^{1}H$ NMR spectrum of compound M1 in $C_2D_2Cl_4$ at room temperature.

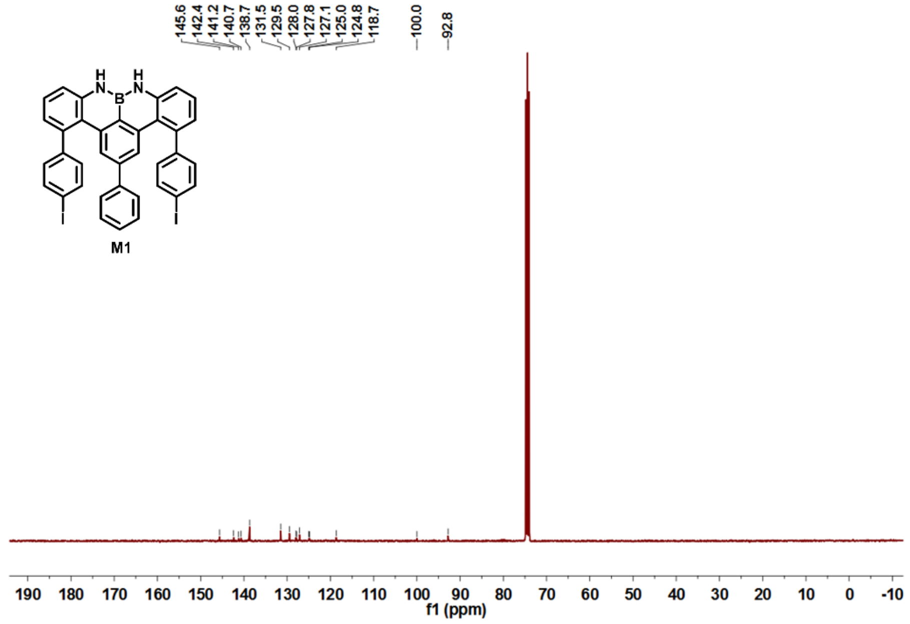

**Figure S39**: 76 MHz $^{13}C$ NMR spectrum of compound M1 in $C_2D_2Cl_4$ at room temperature.

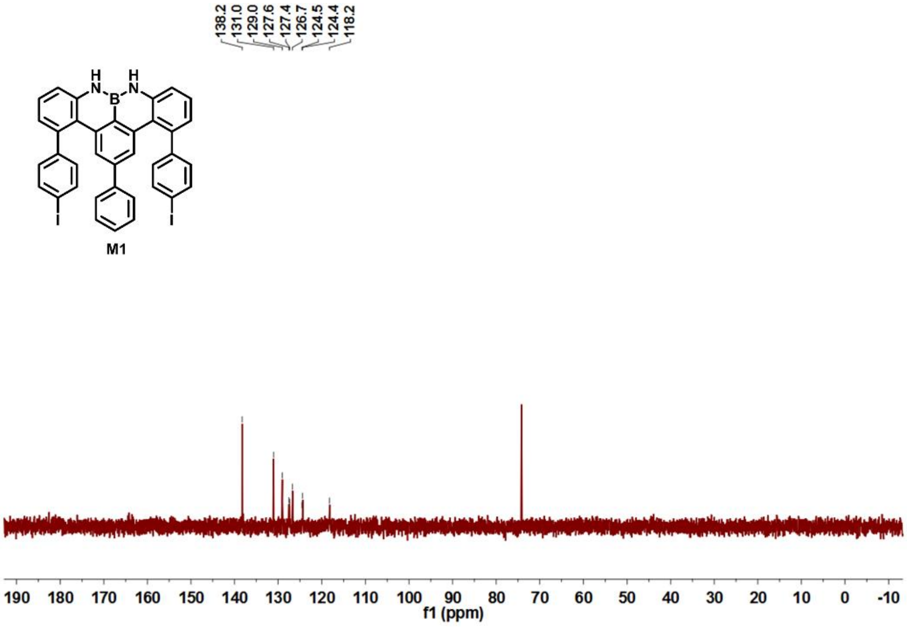


**Figure S40**: 76 MHz $^{13}$C-DEPT-135 NMR spectrum of compound M1 in $C_2D_2Cl_4$ at room temperature.

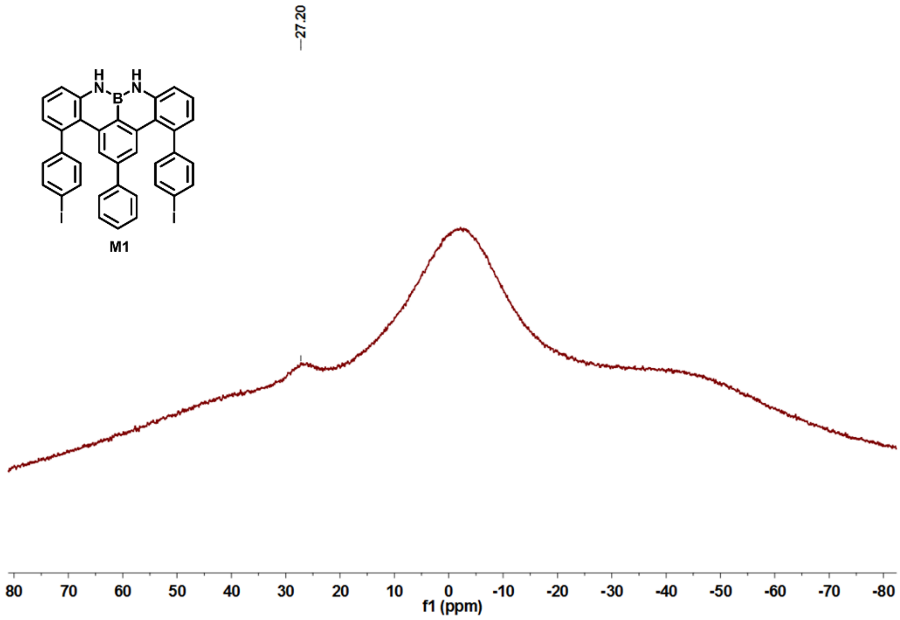


**Figure S41**: 96 MHz $^{11}$B NMR spectrum of M1 in $C_2D_2Cl_4$ at room temperature.

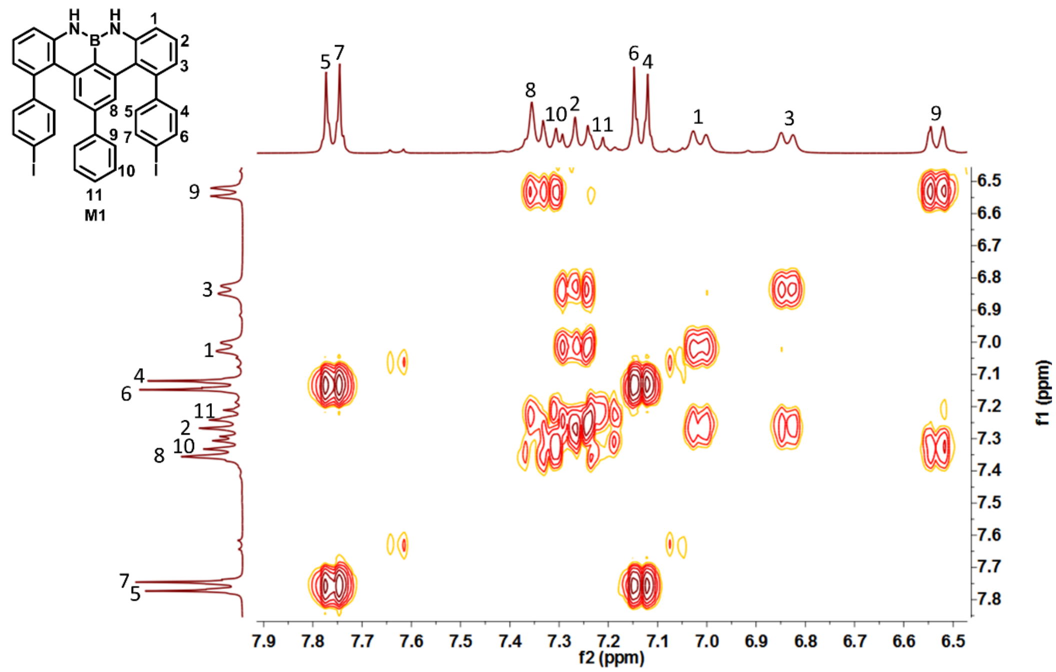

**Figure S42**: 300 MHz $^{1}H/^{1}H$ COSY spectrum of M1 in $C_2D_2Cl_4$ at room temperature.

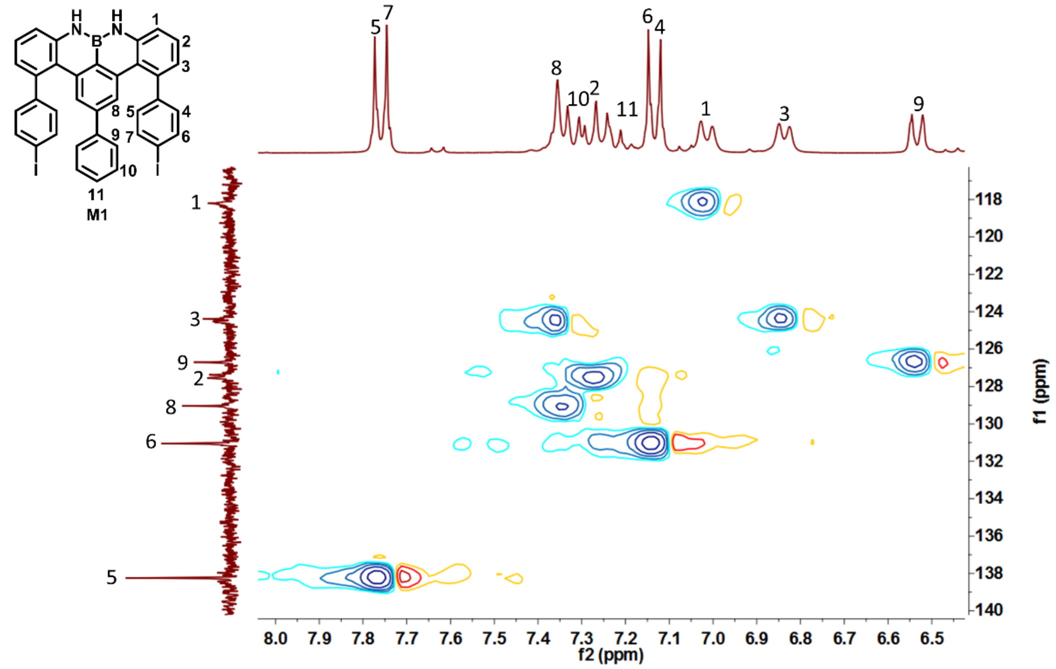

**Figure S43**: 300/76 MHz $^{1}H/^{13}C$-DEPT-135 HSQC spectrum of M1 in $C_2D_2Cl_4$ at room temperature.

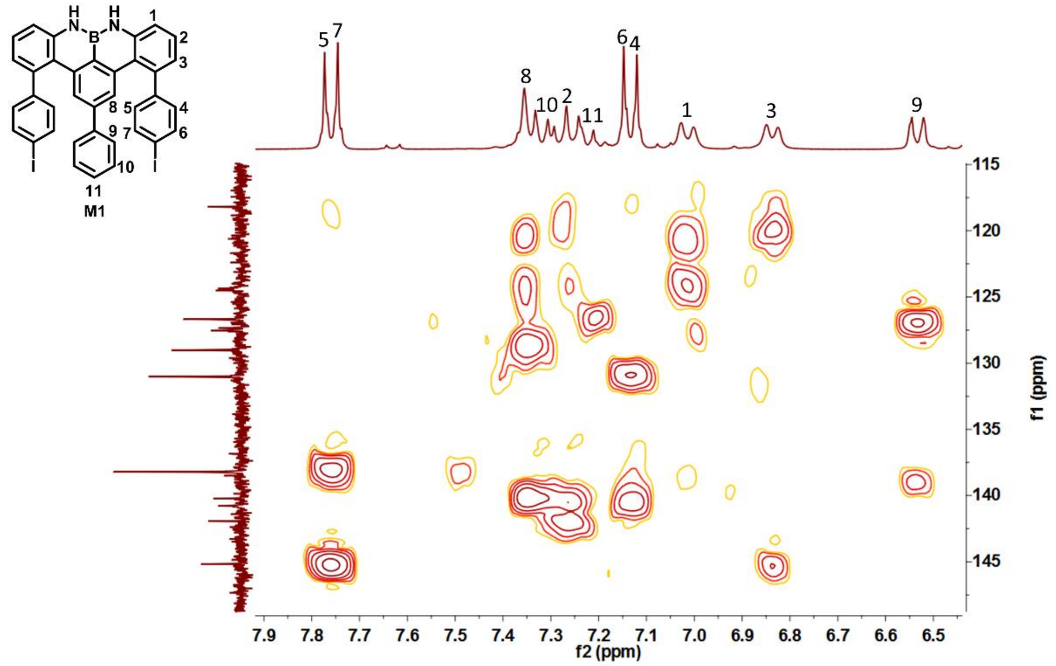


**Figure S44**: 300/76 MHz $^{1}H/^{13}C$ HMBC spectrum of M1 in $C_2D_2Cl_4$ at room temperature.

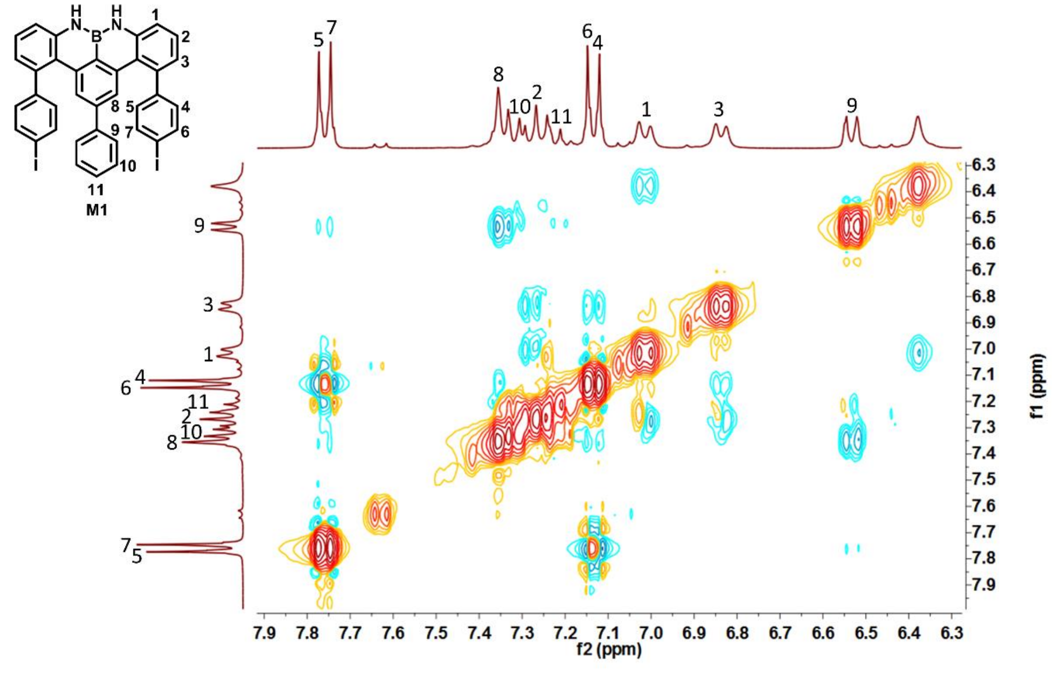


**Figure S45**: 300 MHz $^{1}H/^{1}H$ NOESY spectrum of M1 in $C_2D_2Cl_4$ at room temperature.

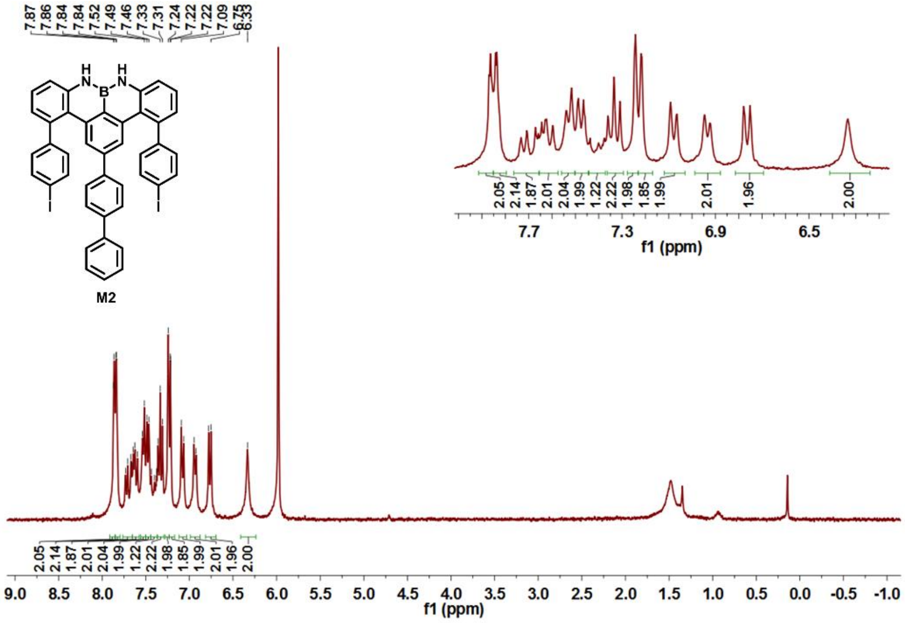


**Figure S46**: 300 MHz $^{1}$H NMR spectrum of compound M2 in $C_2D_2Cl_4$ at 393 K.

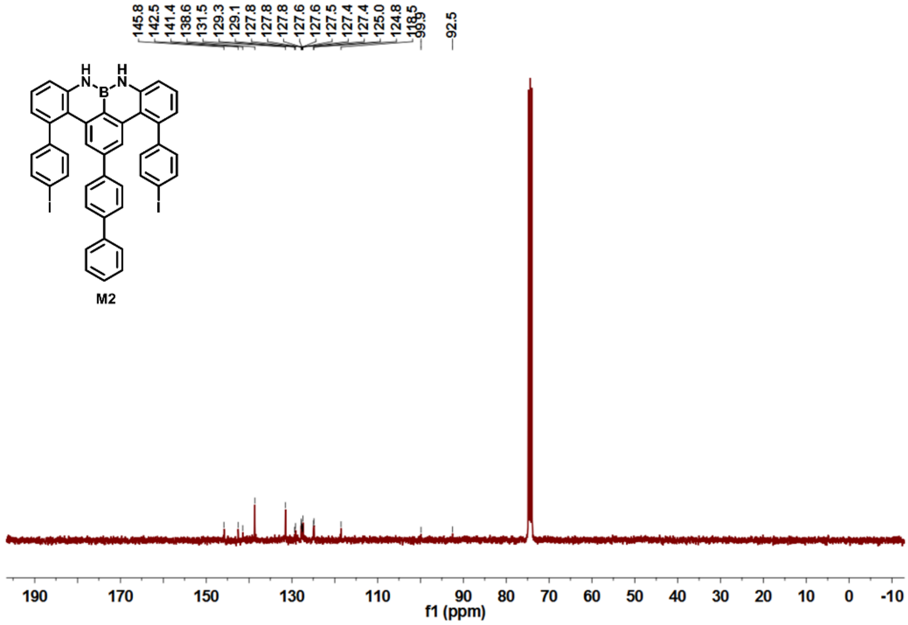


**Figure S47**: 75 MHz $^{13}$C NMR spectrum of compound M2 in $C_2D_2Cl_4$ at 393 K.

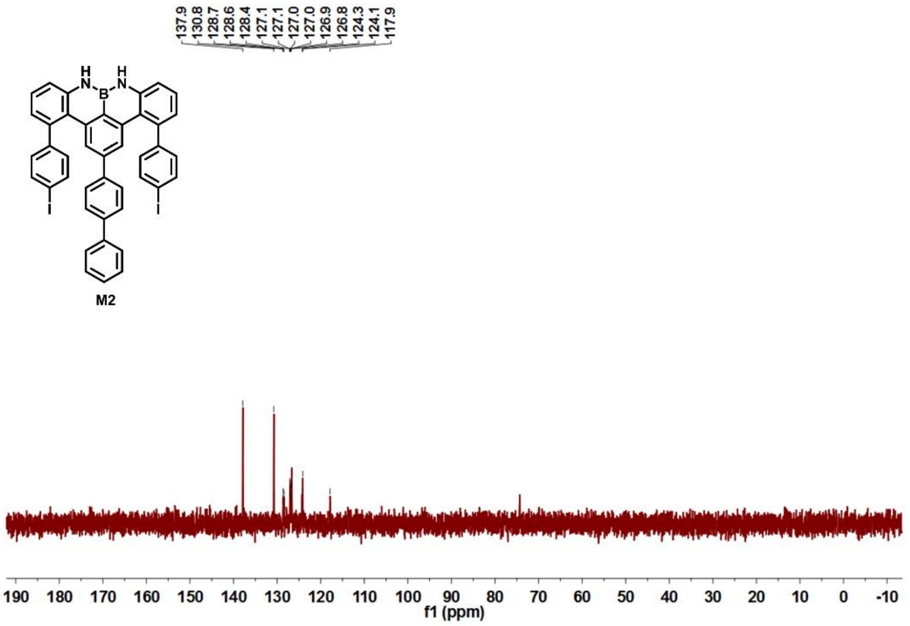


**Figure S48**: 75 MHz $^{13}$C-DEPT-135 NMR spectrum of compound M2 in $C_2D_2Cl_4$ at 393 K.

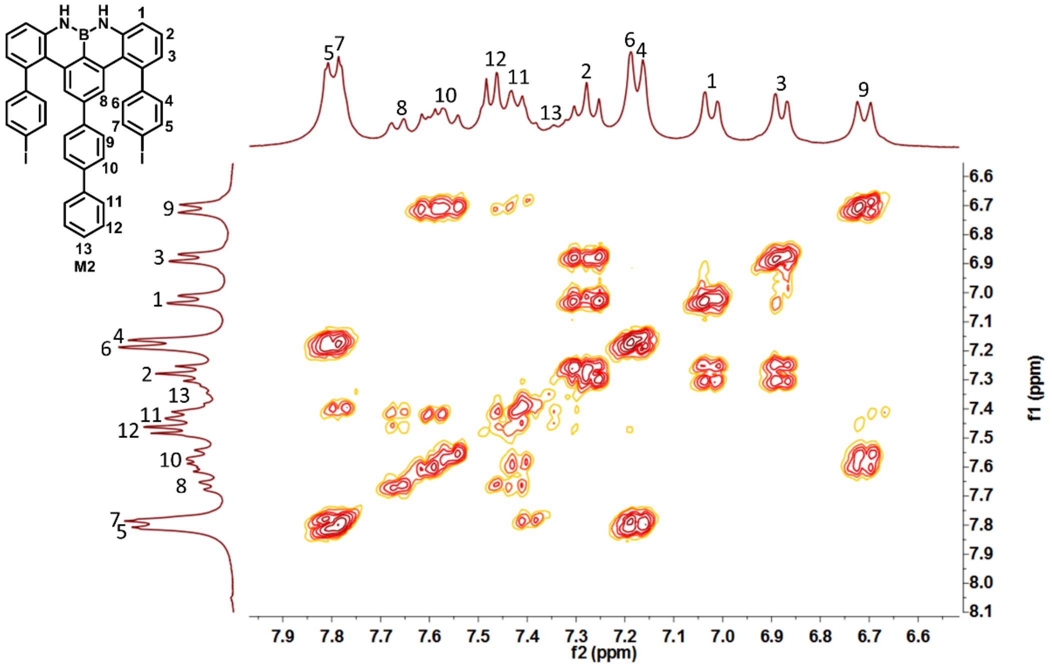


**Figure S49**: 300 MHz $^{1}$H/$^{1}$H COSY spectrum of M2 in $C_2D_2Cl_4$ at 393 K.

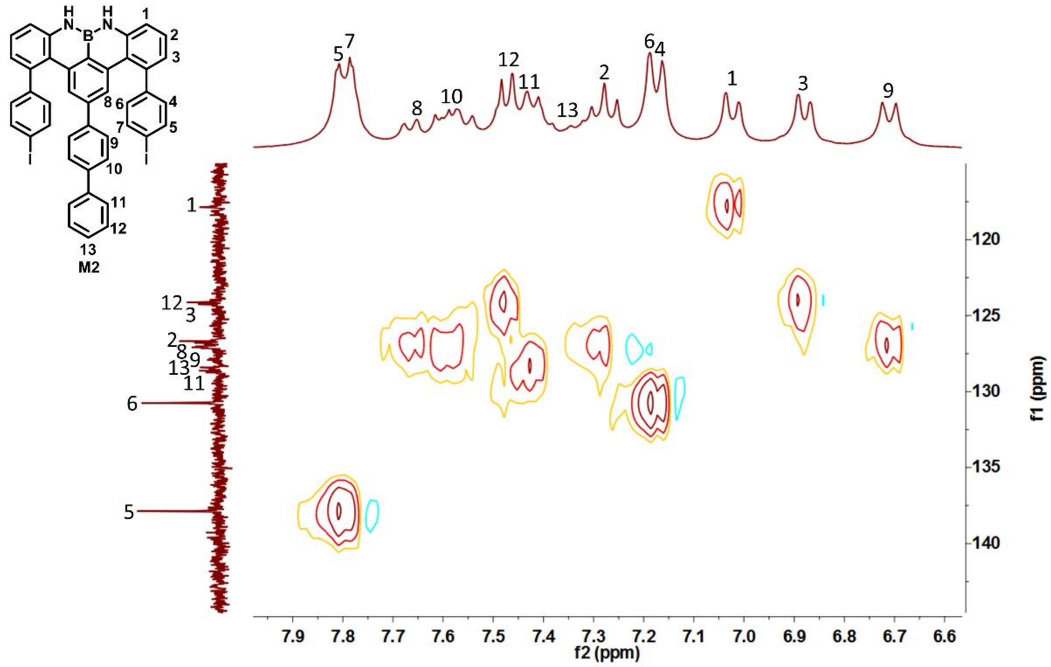


**Figure S50**: 300/76 MHz $^{1}H/^{13}C$-DEPT-135 HSQC spectrum of M2 in $C_2D_2Cl_4$ at 393 K.

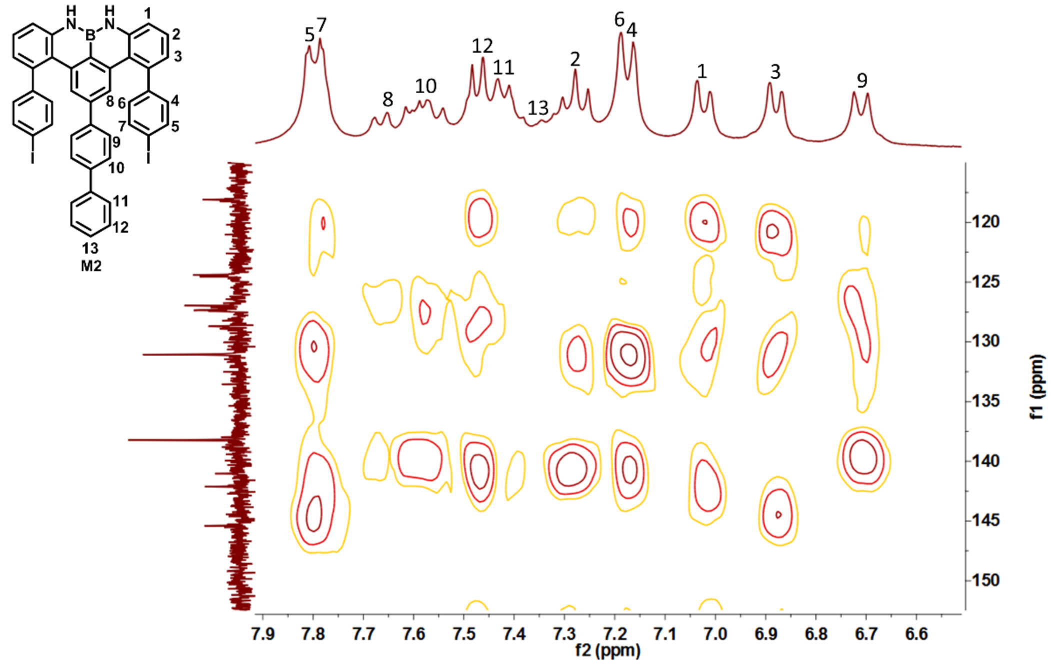


**Figure S51**: 300/76 MHz $^{1}H/^{13}C$ HMBC spectrum of M2 in $C_2D_2Cl_4$ at 393 K.

## 7. High-Resolution Mass Spectroscopy (HRMS)

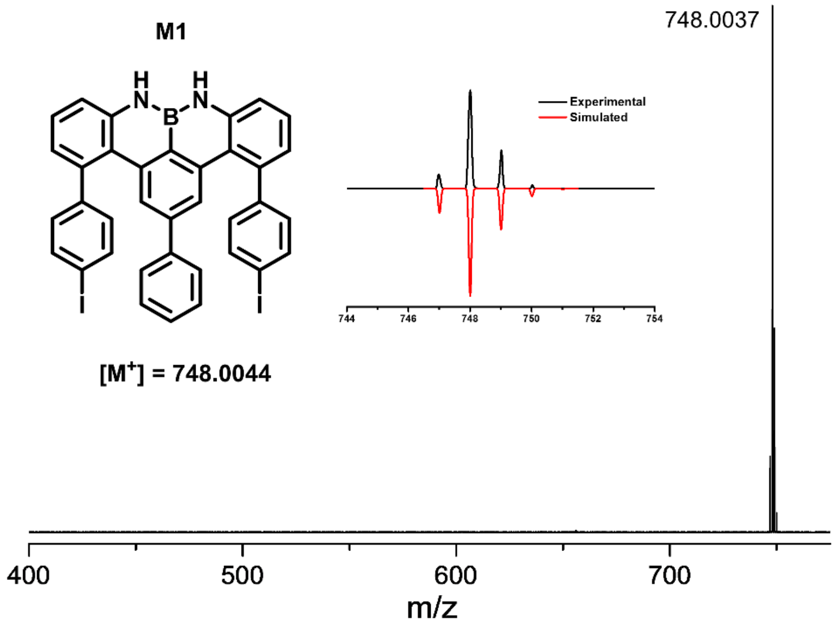


**Figure S52**: HR-MALDI TOF spectrum of M1 in DCTB.

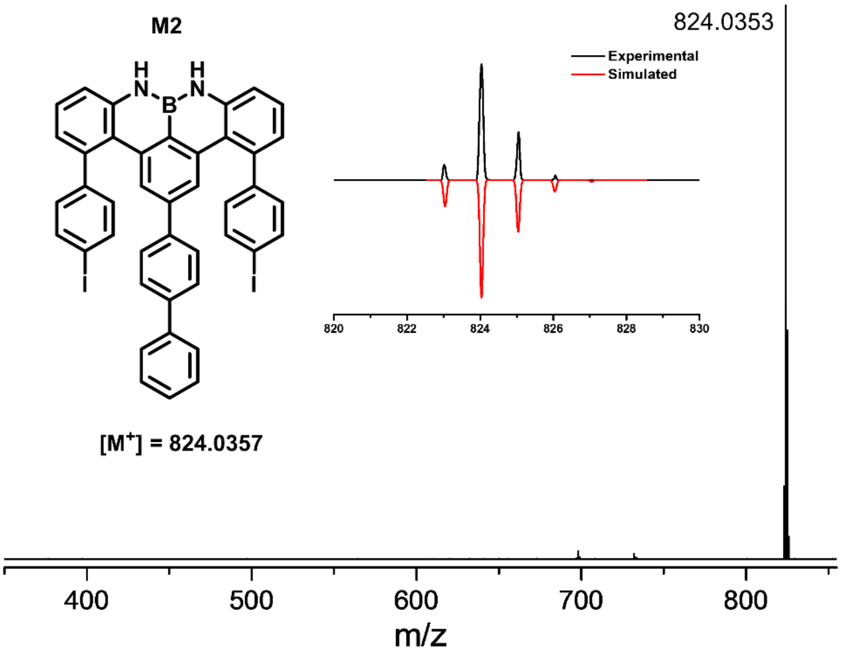


**Figure S53**: HR-MALDI TOF spectrum of M2 in DCTB.

## 8. References

[1] H. D. Thaker, A. Som, F. Ayaz, D. Lui, W. Pan, R. W. Scott, J. Anguita, G. N. Tew, *J. Am. Chem. Soc.* **2012**, *134* (27), 11088–11091.

[2] X. Yan, B. Li, X. Cui, Q Wei, K, Tajima, L. Li, *J. Phys. Chem. Lett.* **2011**, *2*, 10, 1119–1124.

[3] H. Petzold, A. I. Alrawashdeh, S. Heider, L. Haufe, T. Rüffer, *Eur. J. Inorg. Chem.* **2013**, 4858–4866.

[4] F. J. Giessibl, *Rev. Mod. Phys.* **2003**, 75(3), 949-983.

[5] L. Bartels, G. Meyer, K.-H. Rieder, *Appl. Phys. Lett.* **1997**, 71(2), 213-215.

[6] X. Wang, F. Zhang, K. S. Schellhammer, P. Machata, F. Ortmann, G. Cuniberti, Y. Fu, J. Hunger, R. Tang, A. A. Popov, R. Berger, K. Müllen, X. Feng, *J. Am. Chem. Soc.* **2016**, *138* (36), 11606–11615.

[7] Y. Fu, K. Zhang, E. Dmitrieva, F. Liu, J. Ma, J. J. Weigand, A. A. Popov, R. Berger, W. Pisula, J. Liu, X. Feng, *Org. Lett.* **2019**, *21* (5), 1354–1358.

[8] D. Beyer, S. Wang, C. A. Pignedoli, J. Melidonie, B. Yuan, C. Li, J. Wilhelm, P. Ruffieux, R. Berger, K. Müllen, R. Fasel, X. Feng, *J. Am. Chem. Soc.* **2019**, *141* (7), 2843-2846.

[9] J. P. Perdew, K. Burke, M. Ernzerhof, *Phys. Rev. Lett.* **1996**, *77*, 3865–3868.

[10] G. Kresse, J. Furthmüller, *Comp. Mater. Sci.* **1996**, *6*, 15–50.

[11] G. Kresse, J. Furthmüller, *Phys. Rev. B* **1996**, *54*, 11169–11186.
